# MicroRNA biomarkers as next-generation diagnostic tools for neurodegenerative diseases: a comprehensive review

**DOI:** 10.3389/fnmol.2024.1386735

**Published:** 2024-05-31

**Authors:** Hafiz Muhammad Husnain Azam, Rosa Ilse Rößling, Christiane Geithe, Muhammad Moman Khan, Franziska Dinter, Katja Hanack, Harald Prüß, Britta Husse, Dirk Roggenbuck, Peter Schierack, Stefan Rödiger

**Affiliations:** ^1^Institute of Biotechnology, Faculty of Environment and Natural Sciences, Brandenburg University of Technology Cottbus-Senftenberg, Senftenberg, Germany; ^2^German Center for Neurodegenerative Diseases (DZNE), Berlin, Germany; ^3^Department of Neurology, Charité – Universitätsmedizin Berlin, Berlin, Germany; ^4^Faculty of Health Sciences, Joint Faculty of the Brandenburg University of Technology Cottbus – Senftenberg, The Brandenburg Medical School Theodor Fontane and the University of Potsdam, Berlin, Germany; ^5^PolyAn GmbH, Berlin, Germany; ^6^Institute of Biochemistry and Biology, University of Potsdam, Potsdam, Germany

**Keywords:** neurodegenerative diseases, microRNA, biomarkers, nervous system, diagnostic tools, therapeutic tools, protein post-translational modifications, limitations

## Abstract

Neurodegenerative diseases (NDs) are characterized by abnormalities within neurons of the brain or spinal cord that gradually lose function, eventually leading to cell death. Upon examination of affected tissue, pathological changes reveal a loss of synapses, misfolded proteins, and activation of immune cells—all indicative of disease progression—before severe clinical symptoms become apparent. Early detection of NDs is crucial for potentially administering targeted medications that may delay disease advancement. Given their complex pathophysiological features and diverse clinical symptoms, there is a pressing need for sensitive and effective diagnostic methods for NDs. Biomarkers such as microRNAs (miRNAs) have been identified as potential tools for detecting these diseases. We explore the pivotal role of miRNAs in the context of NDs, focusing on Alzheimer’s disease, Parkinson’s disease, Multiple sclerosis, Huntington’s disease, and Amyotrophic Lateral Sclerosis. The review delves into the intricate relationship between aging and NDs, highlighting structural and functional alterations in the aging brain and their implications for disease development. It elucidates how miRNAs and RNA-binding proteins are implicated in the pathogenesis of NDs and underscores the importance of investigating their expression and function in aging. Significantly, miRNAs exert substantial influence on post-translational modifications (PTMs), impacting not just the nervous system but a wide array of tissues and cell types as well. Specific miRNAs have been found to target proteins involved in ubiquitination or de-ubiquitination processes, which play a significant role in regulating protein function and stability. We discuss the link between miRNA, PTM, and NDs. Additionally, the review discusses the significance of miRNAs as biomarkers for early disease detection, offering insights into diagnostic strategies.

## 1 Neurodegenerative disease

This review delves into the significance of microRNAs (miRNAs) involved in neurodegenerative diseases (NDs) in terms of biomarkers contributing to diagnostics. In general, NDs occur due to abnormalities in the neural networks, i.e., structural and functional loss of neurons because of severe damage (Agrawal and Biswas, 2015). Only symptomatic treatment is available for NDs; no drugs or medications can cure or prevent them (Grasso et al., 2014). In our study, we focused on the most prevalent and well-known NDs, such as Alzheimer’s disease (AD), Parkinson’s disease (PD), Multiple Sclerosis (MS), Huntington’s disease (HD), and Amyotrophic Lateral Sclerosis (ALS). Neurological disorders pose substantial global healthcare challenges, as they diminish quality of life and constitute a major health concern. Their prevalence, escalating healthcare costs, and growing incidence in aging populations contribute to this issue. The intricate nature of these conditions complicates diagnosis and treatment, often leading to misdiagnosis and delayed care – exacerbating symptoms and burdening patients and caregivers. To effectively address these challenges, a multifaceted strategy is required: enhancing access to care, investing in research for innovative diagnostic and therapeutic solutions, and raising public awareness about neurological disorders.

Aging is characterized by a gradual decline in decreased efficiency, and impaired tissue and organ function. The decrease in physical well-being increases the probability of death and vulnerability to a range of age-related diseases, including neurological disorders, osteoporosis, sarcopenia, cancer, and cardiovascular diseases (Santos and Lindner, 2017). The most frequent NDs are age-related diseases that cause memory and behavioral impairment (Tan et al., 2015). A United Nations report estimated that 1 in 11 cases of NDs occurred in people over the age of 65 years in 2019 and this would increase to an alarming number of 1 in 6 by the end of 2050 (Zheng and Chen, 2022). Researchers project that dementia cases in the developed world are expected to increase from 13.5 million in 2000 to 21.2 million by 2025. The projected figure is expected to rise to 36.7 million by 2050 (Zheng and Chen, 2022).

As the brain ages, it experiences progressive weight loss, reduced overall myelinated axon length, and diminished cortical volume resulting from neuronal atrophy predominantly affecting the frontal and temporal lobes. These changes are associated with aging-related alterations that may influence the development or progression of NDs, making aging the primary risk factor (Sowell et al., 2004; Hou et al., 2019). Disruption in protein balance, which is prominent in many age-related NDs, including AD and PD, is also widely detected throughout the normal aging process. Older people may show a more significant number of aggregates like β-amyloid (A-β), tau, and α-synuclein, even if they lack any noticeable cognitive problems or disease symptoms (Elobeid et al., 2016; Burdukiewicz et al., 2017). In older people, cognitive function declines, making them more susceptible to NDs (Hedden and Gabrieli, 2004). While metabolic dysfunction, neurite, and synaptic loss are observed in some NDs, such as AD and PD, the specific progression can vary among different conditions (Agrawal, 2020; Lamptey et al., 2022). The transition from metabolic disorders to the loss of neurites and synapses is not a universal feature of all NDs. A further consequence of NDs is that they spread to other brain regions and affect different types of cells (von Bernhardi, 2008). Overall cognitive decline can also lead to a decrease in quality of life as individuals with NDs become increasingly dependent on others for care and support. Additionally, these diseases are costly to treat, damaging healthcare systems. Thus, it is essential to focus on preventive measures to reduce the risk of cognitive decline.

Several studies have shown that neuronal problems often start 10–20 years before symptoms appear. As a result, it can sometimes be detected in its presymptomatic stage by using biomarkers such as serum neurofilament light chain (NfL), which detects neuronal damage before symptoms manifest (Gaetani et al., 2021; Piscopo et al., 2021). To treat these diseases effectively, new tools are needed to identify them as soon as possible, even before symptoms appear. Neurodegenerative diseases are diagnosed using magnetic resonance imaging (MRI), molecular diagnostics, biomarker analysis, and neuroimaging. Several types of dementia can be distinguished using structural MRI scans such as frontotemporal dementia (FTD), vascular dementia, and Lewy body dementia. Moreover, modern methods, such as positron emission tomography (PET) scans and cerebrospinal fluid (CSF) analysis, can also be used to detect NDs in their early stages (Sheinerman and Umansky, 2013; Agrawal and Biswas, 2015; Koikkalainen et al., 2016; Shusharina et al., 2023; Sharma et al., 2024). It has been shown that phosphorescence lifetime imaging microscopy (PLIM), fluorescence lifetime imaging microscopy (FLIM), fluorescence resonance energy transfer (FRET) (Ishikawa-Ankerhold et al., 2012; Jahn et al., 2015), and dot-blot (Subramanian et al., 2016) can improve diagnostics. In addition to measuring protein degradation, these techniques can detect CSF substances. Although these methods are less intrusive and costly, they are not ideal for initial screening (Apostolova et al., 2010; Fagan et al., 2011; Mori et al., 2012).

The study of NDs contributes to a better understanding of the general mechanisms of neurodegeneration. In the future, these findings can lead to the development of new biomarkers, diagnostic tools, and therapies. In this review, we will focus on molecular methods, specifically regarding the application of miRNAs as biomarkers for early disease detection. NDs can be diagnosed and detected using molecular diagnostics, crucial for accurately identifying and stratifying patients, which is vital for developing effective treatments and enhancing our understanding of these complex diseases. We also explore the aging process and its association with various molecular and cellular changes, including changes in miRNA expression and RNA-binding proteins (RBPs), both implicated in ND pathogenesis.

## 2 Biomarkers for neurodegenerative diseases

**Figure 1:**
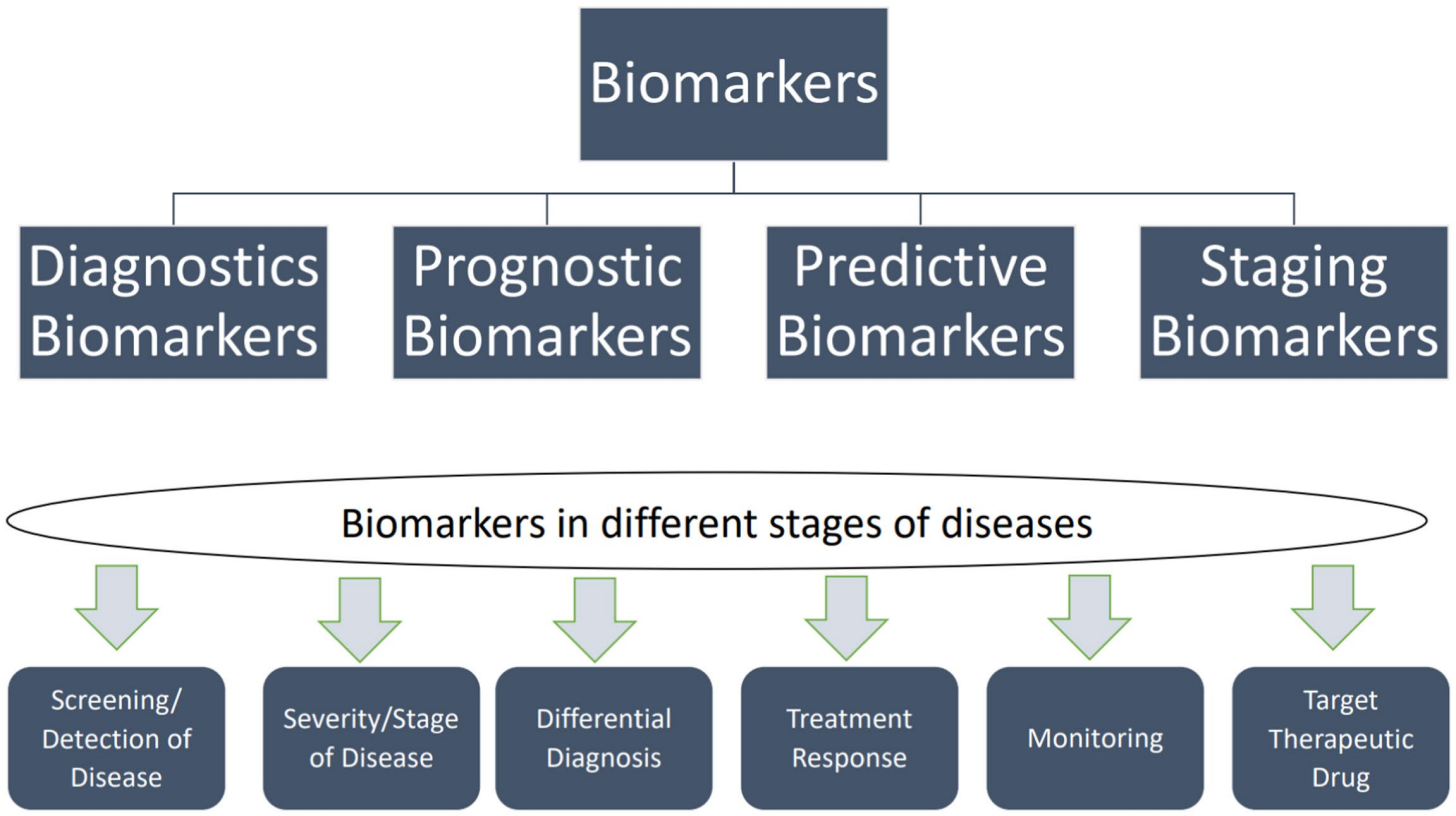
Types of biomarkers and their uses in different disease stages. Biomarkers can be categorized into two main types: exposure biomarkers and disease biomarkers. Exposure biomarkers are employed to predict the occurrence of a disease, while disease biomarkers are utilized for monitoring, screening, and diagnosing a disease. Moreover, predictive biomarkers can forecast an individual’s vulnerability to disease (Taj et al., 2020).

Biomarkers are traits that can be measured and used to show whether biological processes are objectively normal, whether a response to medication can be expected, or whether pathogenic processes are taking place so that the right treatments can be used. Figure 1 illustrates the applications of biomarkers at different disease stages. Molecular diagnostics can identify them using neuroimaging techniques such as PET, MRI, and nuclear magnetic resonance spectroscopy (NMRS) (Rachakonda et al., 2004; Agrawal and Biswas, 2015; Hussain et al., 2022). The combination of biomarkers and NMRS has demonstrated promise in molecular diagnostics alongside other techniques such as diffusion tensor imaging (DTI), functional MRI, and PET. NMRS is a quantitative imaging method that allows researchers to use specific neuronal metabolites as biomarkers to study real-time metabolic dysfunction and permanent neuronal damage. Researchers investigated whether NMRS could serve as a molecular imaging biomarker for *in vivo* diagnosis of PD and monitoring treatment efficacy (Ciurleo et al., 2014).

### 2.1 Fundamentals of microRNA

The sequencing of the human genome revealed a surprising finding. Only a relatively small part of the genetic information is coding for proteins. It is widely believed that a significant portion of the human genome, estimated at 90% by educated guesses, is non-coding (Santosh et al., 2015). Initially, it was assumed that the non-coding part was junk DNA. However, research has shown that non-coding DNA is constantly transcribed into non-coding RNA. These RNA molecules have specific physiological functions in cells (Santosh et al., 2015). Among the most critical classes of RNA are miRNAs and small interfering RNAs (siRNAs), which are typically 20 to 30 nucleotides in length, play a key role in regulating gene expression, and are implicated in diseases including NDs and cancer. While structurally and functionally similar, there are crucial differences between siRNAs and miRNAs. MicroRNAs are single-stranded, endogenously derived from non-coding RNA, while siRNAs are exogenous double-stranded RNA taken up by cells. Therefore, they have distinct properties that make them useful for different purposes.

Additionally, siRNAs are specific for a single target mRNA. At the same time, miRNAs can have multiple mRNA targets (exact numbers can vary widely depending on the particular miRNA and cellular context) due to their imperfect pairing. These differences contribute to their distinct roles in gene regulation and potential therapeutic applications (Mattick and Makunin, 2006; Esteller, 2011; Hombach and Kretz, 2016; Yang et al., 2016).

Mature miRNAs are made up of 21–25 nucleotide sequences. They are the end products of non-protein-coding genes and are known to be the most abundant class of RNA molecules (> 25,000 evolutionarily distinct miRNAs) in animals and plants (Chen, 2005; Grasso et al., 2014). MicroRNAs regulate 30% of human genes and are involved in the post-transcriptional regulation of gene expression linked to regulating many protein-coding genes (Li et al., 2023).

#### 2.1.1 Biogenesis of microRNA

The biogenesis of miRNAs follows two pathways: (a) the intergenic (canonical processing) pathway and (b) the intronic (non-canonical) pathway (Bartel, 2004; MacFarlane and Murphy, 2010; Miyoshi et al., 2010; Ha and Kim, 2014; O’Brien et al., 2018).

**Figure 2:**
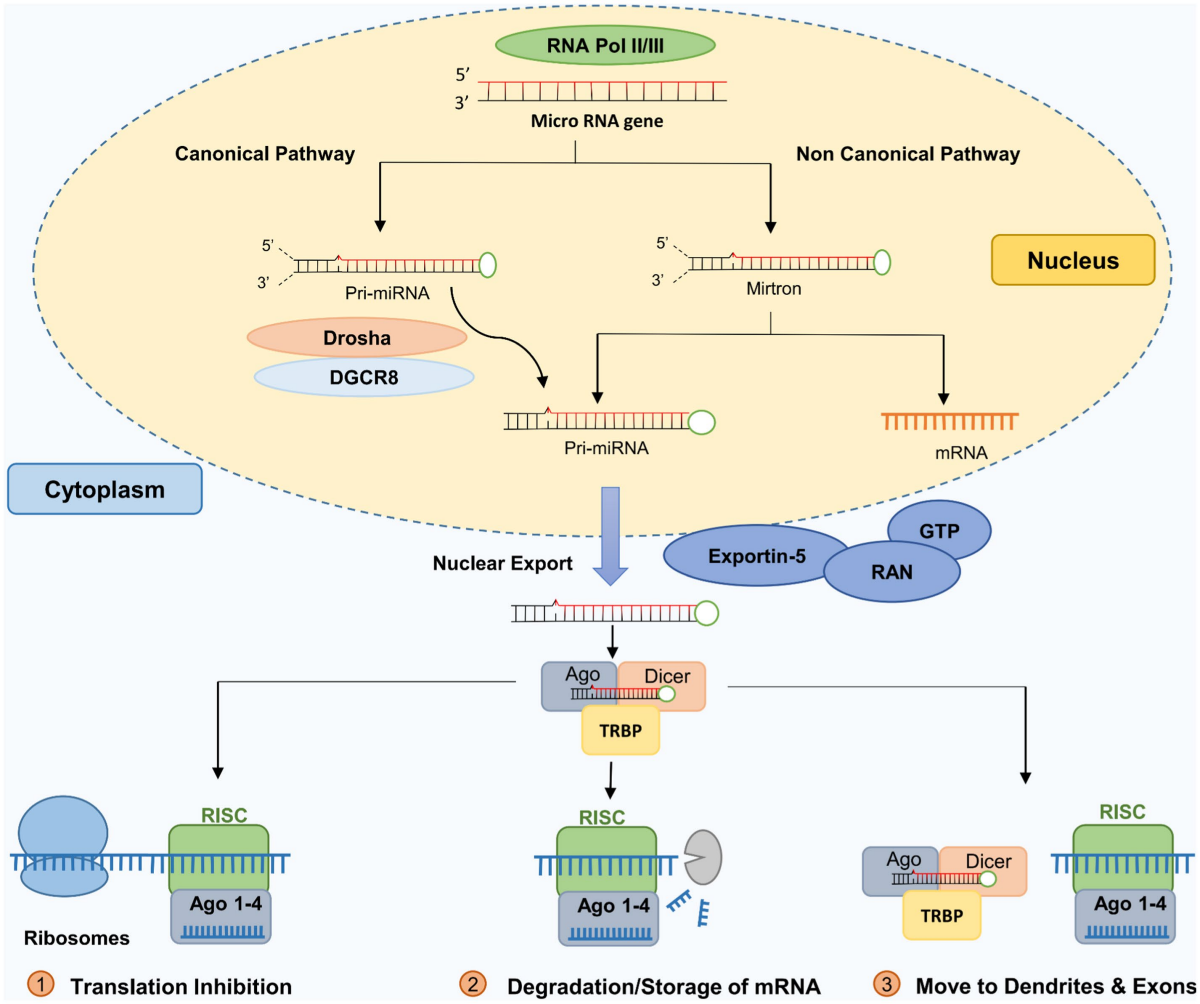
MicroRNA biosynthesis pathways. Adapted from Huang (2022) by biorender.com. For details, see (O’Carroll, D., et al., 2013; Maffioletti, E., et al., 2014).

The canonical miRNA biogenesis process (Intergenic miRNA) involves the two RNase III-type proteins, Drosha and Dicer. It begins with RNA-Polymerase II transcription of the primary miRNA-transcripts (pri-miRNAs). A microprocessor complex containing Drosha and DGCR8 (DiGeorge syndrome critical region 8) cleaves primary miRNA transcripts to produce miRNA precursor molecules (pre-miRNA). A pronounced hairpin structure characterizes pre-miRNAs. The pre-miRNAs are transported into the cytoplasm and cleaved by the RNase III enzyme Dicer into 20–23 nucleotides dsRNAs. The short-lived dsRNAs are unwound and then become single-stranded miRNAs. Mature miRNA is then incorporated into specific protein complexes, referred to as micro-ribonucleoprotein complexes (miRNPs) (Gregory et al., 2006; Beezhold et al., 2010; MacFarlane and Murphy, 2010; Wahid et al., 2010). Pre-miRNAs are transcribed into miRNAs within the nucleus by RNA polymerase II (Chen, 2005). Two RNA III enzymes then process this miRNA precursor (Wu et al., 2012). Drosha, DGCR8 (DiGeorge Critical Region 8), and the RNase III enzyme (RNASEN) form a protein complex after the first transcription. Figure 2 shows that a protein cooperating with Drosha and other helpers locates the primary miRNA and truncates its ends to generate the premature miRNA (Lee et al., 2003; Han et al., 2004). Exportin-5 is crucial for the pre-miRNA to be targeted and transported to the cytosol, where it is further processed via the GTP-binding nuclear protein Ran (RanGTP)-dependent pathway (Yi et al., 2003; Bohnsack et al., 2004). The Dicer complex, consisting of Argonaute RNA-induced silencing complex (RISC) catalytic component 2 (AGO2), Dicer (RNase), protein activator of the interferon-induced protein kinase (PACT), and transactivation response element RNA-Binding Protein (TRBP), transforms pre-miRNAs in the cytosol into mature miRNA duplexes composed of 22 nucleotides (Gregory et al., 2004; MacRae et al., 2008). Dicer is responsible for separating two types of miRNA, the passenger and guide strands. The guide strand communicates with AGO2 in RISC to find the target transcript (Kobayashi and Tomari, 2016).

Non-canonical processing (intronic pathways) refers to alternative pathways for miRNA biogenesis that do not involve Drosha or Dicer. In addition to Drosha/DGCR8 and Dicer, non-canonical miRNA biogenesis involves a variety of proteins that are an integral part of the conventional pathway, such as exportin 5, exportin 6, and AGO2 (MacFarlane and Murphy, 2010). Non-canonical processing pathways are less understood than the canonical pathway, and their roles in miRNA biogenesis and gene regulation are still being investigated. RNA-Polymerase II mediates intronic biogenesis. RNA-Polymerase II directly excises the pre-miRNA, the progenitor of miRNA, from introns.

#### 2.1.2 Functions of microRNAs

More than 2,200 miRNA genes in the human genome are known, which regulate a significant portion of gene expression (Ardekani and Naeini, 2010; O’Brien et al., 2018). The precise mechanism of miRNA transcript positioning is not fully understood, but their location can influence miRNA expression in introns, non-protein-coding genes, exons, and proximity to other genes (O’Brien et al., 2018; Shang et al., 2023). It is reported that non-protein coding genes transcribe approximately 50% of the miRNAs expressed in the human genome and are coded in the introns of coding genes (Ardekani and Naeini, 2010). Human diseases develop and progress are impacted by miRNAs, which are also crucial for regulating drug metabolism and immune responses (Wang and Chen, 2021). MicroRNAs regulate cellular functions such as growth, development, differentiation, metabolism, and immune responses. They achieve this by binding to the corresponding target mRNA and inhibiting protein synthesis in the post-transcriptional phase (Johnston and Hobert, 2003; Ambros, 2004; Poy et al., 2004; Filipowicz, 2005; Hatfield et al., 2005; Zhao et al., 2005; O’Brien et al., 2018).

MicroRNAs play a crucial role in gene regulation through two main mechanisms: mRNA cleavage and translational repression. In the process of mRNA cleavage, miRNAs induce RISC-mediated degradation (cleavage) by binding to complementary regions within protein-coding mRNA sequences (Natarajan et al., 2013). This interaction results in the degradation or repression of target mRNA, impacting cellular processes such as cell division and differentiation (Wang, 2014). The key element in this mechanism is the seed region of the miRNA, a specific 6–8 nucleotide sequence that plays a pivotal role in gene silencing (Ying et al., 2008; O’Brien et al., 2018).

Translational repression involves miRNAs binding to the complementary region of the protein-coding sequence, which halts protein production without degrading the mRNA. This process is characterized by limited base-pairing between the miRNA and its target mRNA. MicroRNAs can bind to the 3′ untranslated regions (UTRs) of mRNAs (Bartel, 2004), inhibiting their translation and thereby regulating gene expression at the post-transcriptional level (Quévillon Huberdeau and Simard, 2019).

Therefore, while both mechanisms involve miRNA binding to specific regions of mRNA, they differ in their outcomes: mRNA cleavage leads to degradation or repression of the target mRNA, affecting gene expression at a broader level, whereas translational repression specifically inhibits protein production without altering mRNA stability. These distinct processes highlight the multifaceted role of miRNAs in fine-tuning gene expression for various cellular functions and regulatory pathways. By modulating these diverse mechanisms, miRNAs contribute to a wide range of biological processes, including development, differentiation, and disease progression.

##### 2.1.2.1 Gene activation

Cell survival requires post-transcriptional regulation of gene expression by miRNAs and miRNPs (Vasudevan, 2012; Basak et al., 2016). MicroRNAs act as inhibitory regulators, repressing translation or enhancing target mRNA degradation. However, miRNAs may indirectly promote translation under specific circumstances or in certain cellular environments, although this is not their primary role. Additionally, miRNAs can lower the amount of expressed repressor proteins, which could cause translation to speed up for specific mRNA targets. It is significant to note that miRNAs regulate multiple mRNA expressions, and conversely, numerous miRNAs can target the same mRNA, contributing to complex regulatory networks within cells (Hashimoto et al., 2013).

## 3 MicroRNA as biomarker

MicroRNAs have gained attention as biomarkers in various scientific fields owing to their numerous desirable properties (Pabinger et al., 2014). The ideal biomarker must be particular, sensitive, and predictive (Lu et al., 2005). In body fluids, miRNAs are protected inside exosomes, microvesicles, apoptotic bodies, or protein complexes (bound to AGO2 or RISC). They exhibit stability in various conditions, including room temperature, boiling, multiple freeze-thawing cycles, pH fluctuations, and chemical or enzymatic fragmentation. MicroRNAs have a strong specificity and can be used with formalin-fixed, paraffin-embedded tissues (FFPE) and fresh, snap-frozen samples (Xi et al., 2007). This stability is essential for the clinical application of miRNA biomarkers because, in the context of pre-analytical procedures, it allows for easy transportation of samples to the clinical lab without needing special handling or immediate processing. Moreover, the pre-analytical phase is prone to less error, which is crucial for accurate and reliable results (Mitchell et al., 2008; Cortez et al., 2011; Mo et al., 2012).

Several body fluids, including blood, saliva, plasma, tears, and CSF, can measure miRNAs (Weber et al., 2010). A variety of techniques have been developed to profile miRNAs (Wang B. et al., 2011), including microarrays, sequencing, and quantitative reverse transcription PCR (RT-qPCR). Rio et al. (2010) purified the sample from total RNA using tri-reagents of acid phenol and guanidinium-thiocyanate, column filtration protocols, and chloroform from total RNA. It is widely accepted that adopting an appropriate normalized approach is critical to eliminate variances and improve the accuracy of miRNA quantification (Rio et al., 2010).

In general, there are no significant variations in the extraction of miRNAs from serum and venous plasma. However, sampling studies on humans have concluded that researchers should carefully select the blood collection procedure for specific miRNA biomarkers (Zhou et al., 2016). The detection of miRNAs molecules is difficult due to their inherent properties, such as their tiny size, low level, sequence homology across members, and tissue-or stage-specific expression (Wang J. et al., 2016; Ricci et al., 2018; Felekkis and Papaneophytou, 2020; Djebbi et al., 2021). Earlier research on miRNAs in the bloodstream failed to develop accurate biomarkers. Overlooking factors such as previous treatments, age, and gender contribute to this problem. Ensuring the reliability of both positive and negative results is challenging. Minimizing experimental or technical variations is crucial due to the minor differences in miRNA expression levels observed between healthy and sick persons. Researchers should carefully control the steps for miRNA identification, data processing, data normalization, and data optimization (Pabinger et al., 2014; Witwer, 2015). In this regard, a mix of exogenous and endogenous control miRNAs is recommended, as a single reference gene has proven unreliable (Schwarzenbach et al., 2015).

Evidence shows that miRNAs can be used as diagnostic biomarkers for various diseases. MicroRNAs have limited clinical applications due to inconsistencies in the literature, standard assays, and a lack of reproducibility (Precazzini et al., 2021; Ho et al., 2022). Additionally, selecting a suitable reference for miRNA quantification in biofluids is problematic. There is currently no method of analyzing circulating miRNAs without RNA extraction and purification, which may result in miRNA loss (Precazzini et al., 2021). Despite these challenges, circulating miRNAs have been established as promising non-invasive biomarkers for accurate diagnosis, prognosis of disease progression, and treatment responsiveness (Condrat et al., 2020). At least for cancer and other diseases, several miRNAs panels are already available for clinical use, and they have shown potential for early detection, subtype classification, and treatment strategy selection in cancer and other diseases (Wang R. et al., 2018; Tribolet et al., 2020). Another challenge is that miRNAs’ functionality can vary by disease stage, making them difficult to use as specific targets (Cao et al., 2016). Thus, circulating miRNAs as biomarkers still requires further research and multiple independent validation studies for clinical application.

### 3.1 MicroRNA in the brain

MicroRNA expression levels change during brain development, with different miRNAs expressed at different stages (Cho et al., 2019; Prodromidou and Matsas, 2019; Brennan and Henshall, 2020). High-throughput sequencing analyses show that the human brain expresses at least 550 miRNAs, a higher expression level than in other organs (Ardekani and Naeini, 2010; Chen and Qin, 2015).

MicroRNAs are crucial brain regulators and frequently show brain-specific expression patterns. MicroRNAs commonly co-express with their targets. They are essential in controlling various biological processes, such as synaptic plasticity and neurogenesis (Cao et al., 2016). The miR-134, miR-124, miR-79, miR-9, miR-137, and miR-132 play crucial roles in the growth and development of neurons, synapses, neuroplasticity, and dendrite spine formation (Bandiera et al., 2013; Sharma and Lu, 2018; Paul et al., 2020). A study demonstrated that miR-124 expression resulted in a substantial 50% decrease in dendritic spine formation (Garcia et al., 2021). Dysregulation of miRNAs has been observed in several NDs (Grasso et al., 2014), with common pathophysiological characteristics including neuroinflammation, protein aggregation, and mitochondrial dysfunction identified across these disorders. *In vitro* and *in vivo* model systems have been used to explore miRNA functions recently, and as a result, miRNA biomarkers, miRNA regulatory networks, and miRNA pathways have been discovered (Gascon and Gao, 2012; Mo, 2012; Juźwik et al., 2019).

### 3.2 MicroRNA in body fluids

Blood samples have various benefits when used in diagnostic procedures. Blood samples are less invasive to collect and more uncomplicated to store than CSF and tissue biopsies. Since miRNAs are stable in the blood, multiple blood samples can be taken to track the progress of the disease. This boosts the flexibility of the analysis (Ricci et al., 2018). In general, studies showed miRNAs with nominally significant blood–brain correlations. Some have been implicated as peripheral biomarkers of various psychiatric or brain-related disorders (Kos et al., 2022). The use of blood-based miRNAs as a biomarker for ND diagnostics is a topic of ongoing research. Dysregulated miRNAs in the blood have been proposed as potential biomarkers for diagnosing NDs (Mushtaq et al., 2016; Gentile et al., 2022). While miRNAs in the blood have the potential to serve as correlates of brain-based miRNA expression and as biomarkers for NDs, their utility is also influenced by various factors. Biomarkers in the CNS and the blood are regulated by the blood-CSF barrier, which means the same biomarker may be present in both biofluids (Gaetani et al., 2020; Gigase et al., 2023). In addition to nerve damage, neurological disorders often affect other organs and tissues, such as peripheral blood cells and degenerating muscles. In light of this, blood may be a valuable biofluid for discovering and validating ND biomarkers. MicroRNAs in the blood are a limited biomarker for NDs (Kos et al., 2022). MicroRNAs in the blood may indicate inflammatory status, responsiveness to pharmaceutical therapies, or other circumstances not directly connected to an ND. Therefore, they may function as confounding factors (Grasso et al., 2014; Spector et al., 2015; Vu and Bowser, 2017).

MicroRNAs found in the CSF can serve as sensitive indicators of brain changes (Cogswell et al., 2008; Rao et al., 2013) since they indicate both healthy and unhealthy conditions in the CNS. Immune cells, neurons, glial cells, and others secrete miRNAs into the CSF. Macrophages secrete miRNAs into the CSF as part of their physiological activities (Alexandrov et al., 2012; Mori et al., 2019). A study has shown that miRNA levels differ between AD patients with inflammation and non-inflammatory neurological conditions and those with FTD in the serum and cerebrospinal cortex. As compared to non-inflammatory controls, miR-26b (down) and miR-125b were found to be regulated in AD patients’ CSFs (Galimberti et al., 2014; Swarbrick et al., 2019; Noor Eddin et al., 2023).

## 4 MicroRNA biomarkers in neurodegenerative diseases

### 4.1 MicroRNA biomarkers in Alzheimer’s disease

Alzheimer’s disease is an ND that primarily affects older individuals and is becoming a global health concern due to the aging population (Ramachandran et al., 2021). According to statistics from 2020, nearly 55 million people worldwide suffer from AD (Weidner and Barbarino, 2019), and this number is expected to increase by 131.1 million by 2050 (Prince et al., 2015; Nagaraj et al., 2021). The main clinical signs of ADs include memory loss, executive dysfunction, difficulties carrying out routine activities, and visuospatial impairments. Early symptoms of AD include changes in cognitive function, memory loss, and disruptions in language and speech patterns (Tarawneh and Holtzman, 2012). Around 20 to 30% of individuals in the early stages of AD have notable symptoms of sadness and mood alterations, occurring before the first indication of memory decline (Zubenko et al., 2003). Patients in the later stages of AD have severe hallucinations, confusion, and a lack of self-sufficiency, finally dying due to respiratory infection or malnutrition (Kalia, 2003; Tarawneh and Holtzman, 2012). Cerebrovascular amyloidosis, inflammation, and substantial synaptic alterations (Dansokho and Heneka, 2018; Katsumoto et al., 2018) accompany the primary pathological symptoms of AD, which include amyloid beta plaques, gliosis, neurofibrillary tangles (NFTs), and neuronal loss (Itagaki et al., 1989; Terry et al., 1991; Iqbal and Grundke-Iqbal, 2002; Iqbal et al., 2016; Petrella et al., 2019).

**Figure 3:**
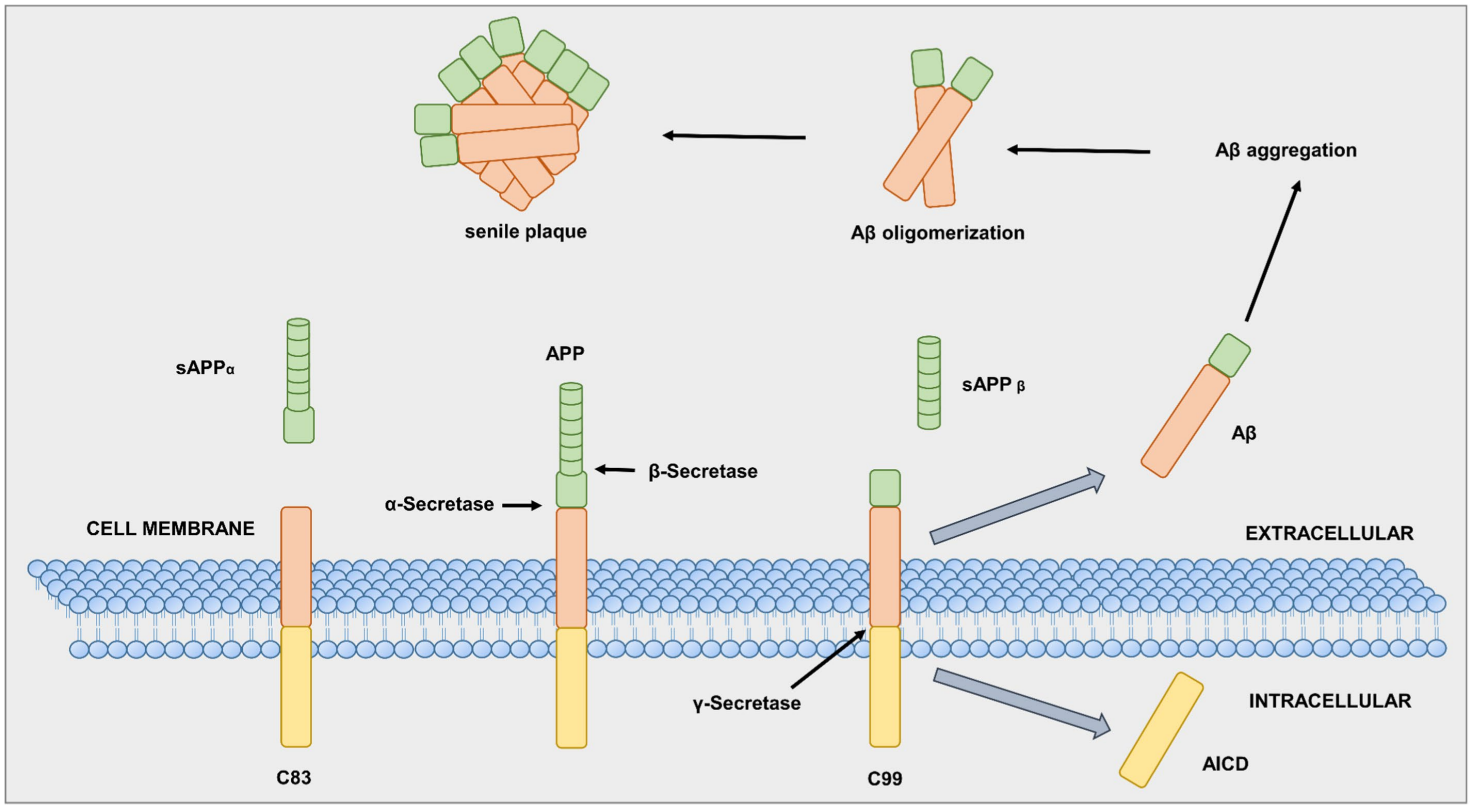
Alpha (α), beta (β), gamma (γ)-secretase in APP processing. Adapted from Gandy S. (2005).

In AD patients, specific brain areas show an accumulation of two abnormal protein structures, amyloid plaques and NFTs (aggregates of hyperphosphorylated tau protein in the brain) (Braak and Del Tredici, 2010), along with a loss of cell connections. Both amyloid plaques and NFTs are associated with normal aging; however, in AD patients, these two neuropathological biomarkers are abnormally abundant (Zetterberg and Blennow, 2006). The first source of amyloid plaques is an integral membrane protein, the so-called amyloid-beta precursor protein (APP). The three enzymes alpha (α), beta (β), and gamma (γ) -secretase are responsible for APP cleavage (Epis et al., 2012). First, α-secretase breaks the APP at a crucial point, which prevents amyloid plaques, as shown in Figure 3 (Schedin-Weiss et al., 2014). In contrast, an aspartyl protease called β-secretase, also known as β-site amyloid precursor protein-cleaving enzyme 1 (BACE-1), can attach to the APP cell membrane. The β-secretase cuts the APP protein at one end of the Aβ fragment (O’Brien and Wong, 2011; Simunkova et al., 2019). The remaining piece of APP, previously bound to the neuronal cell membrane, is cleaved by γ-secretase at the other end, resulting in the Aβ protein (MacLeod et al., 2015). Neurons typically produce Aβ protein, which they release into the extracellular space. In general, microglia and astrocytes break down the Aβ protein. However, excess extracellular release of Aβ leads to aggregates of different sizes, called oligomers (Epis et al., 2012; Guo et al., 2020).

Alzheimer’s disease is associated with tau protein, a primary component of NFTs (Kumar et al., 2018; Errico and Meyer-Luehmann, 2020). The tau protein is another abnormal protein structure that accumulates in AD and binds to microtubules in neurons, facilitating axon development. Despite being a natively unfolded protein, tau is highly soluble and does not tend to aggregate easily (Wang and Mandelkow, 2016). AD involves tau protein, the primary component of NFTs (Kumar et al., 2018; Errico and Meyer-Luehmann, 2020). It can undergo hyperphosphorylation in the brain (Iqbal et al., 2016), which leads to the detachment of tau from microtubules, instability of microtubules, self-aggregation, and the formation of neurofibrillary tangles. Several protein kinases and phosphatases regulate tau phosphorylation via phosphorylation-site-dependent pathways.

Furthermore, tau acetylation (Ac-Tau) is associated with tau aggregation, suggesting that Ac-Tau contributes to tau’s pathological conversion. Hence, targeting Ac-Tau may be a promising strategy to prevent tau aggregation and tauopathy progression. The combined effect of tau and Aβ may lead to neurodegeneration in AD. As a result, studies are underway to develop drugs that target Ac-Tau and potentially prevent or slow tauopathy progression. The synergistic interaction between tau and Aβ may cause neurodegeneration in AD (Iqbal et al., 2016; Guo et al., 2020).

In 2007, Schipper and colleagues published their first miRNA biomarker study for AD using microarray analysis. They found that the miRNA expression of peripheral blood mononuclear cells (PBMCs) was higher in AD patients than in healthy cells (Schipper et al., 2007). A 12-miRNA pattern distinguishing between AD patients and healthy blood cells has been identified with next-generation sequencing (NGS). It has 93% accuracy, 95% specificity, and 92% sensitivity (Leidinger et al., 2013).

Kumar et al. (2013) used nanoString technology to detect seven circulating miRNA signals in plasma to distinguish Alzheimer’s patients from healthy controls with 95% accuracy (Kumar et al., 2013). Cogswell and colleagues used RT-qPCR to identify a group of miRNAs only associated with AD. Their miRNA expression patterns in CSF were similar to those in AD patients’ brains (Cogswell et al., 2008). In their study, Garcia et al. (2021) reported the upregulation of miR-21-5p in CSF samples from mild cognitive impairment (MCI) patients who met the criteria for AD, compared to those without AD criteria. This upregulation was also observed in neurons, astrocytes, and microglia derived from induced pluripotent stem cells (iPSCs) of AD patients (Garcia et al., 2021).

Various miRNA-based markers have been identified, showing favorable accuracy, sensitivity, specificity, and cost-effectiveness (Sempere et al., 2004). Table 1 presents an overview of significant miRNAs proposed as AD biomarkers. These miRNAs are thought to be involved in AD pathogenesis and represent a valuable diagnostic tool for early AD diagnosis. Altogether, miRNAs can potentially be a powerful tool in AD diagnosis.

**Table 1:** List of reported miRNAs in Alzheimer’s disease.

Source	miRNA	Expression	Methods	References
Serum	miR-135a, miR-384	Upregulated	RT-qPCR	Yang et al. (2018)
	miR-9, miR-137, miR-181c, miR-29a, miR-29b	Downregulated	RT-qPCR	Geekiyanage et al. (2012)
	miR-193b	Downregulated	RT-qPCR	Yang et al. (2018)
	miR-223-3p	Downregulated	RT-qPCR	Mancuso et al. (2019)
Plasma	miR-486-5p, miR-483-5p	Upregulated	RT-qPCR	Nagaraj et al. (2017)
	miR-15b-5p, miR-191-5p, miR-545-3p, miR-142-3p, let-7 g-5p, miR-301a-3p, let-7d-5p	Downregulated	RT-qPCR, Nanostring	Kumar et al. (2013)
	miR-146a, miR-34a	Downregulated	RT-qPCR	Kiko et al. (2014)
Blood	miR-181b, miR-34a	Upregulated	Microarray, RT-qPCR	Schipper et al. (2007)
Peripheral blood	let-7d-3p, miR-112, miR-26a-5p, miR-5010-3p, miR-151a-3p, miR-1285-5p, miR-161	Upregulated	NGS, RT-qPCR	Leidinger et al. (2013)
	miR-128	Upregulated	RT-qPCR	Tiribuzi et al. (2014)
	miR-532-5p, let-7f-5p, miR-26b-5p, miR-107, miR-103a-3p	Downregulated	NGS, RT-qPCR	Leidinger et al. (2013)
Cerebrospinal fluid	let-7b	Upregulated	TaqMan assay, RT-qPCR	Lehmann et al. (2012)
	miR-9, miR-155, miR-146a, miR-125b	Upregulated	Microarray, LED-Northern dot blot analysis, RT-qPCR	Alexandrov et al. (2012)
	miR-29b, miR-29a	Upregulated	RT-qPCR	Kiko et al. (2014)
	miR-21-5p	Upregulated	RT-qPCR	Garcia et al. (2021)
	miR-448, let-7f, miR-526a, miR-105, miR-520a, miR-518f, miR-374, miR-380-3p, miR-125a, miR-518b, miR-371, miR-135a, miR-517b, miR-138, miR-141, miR-151, miR-30c, miR-517, miR-186, miR-362, miR-501, miR-191, miR-197, miR-494, miR-204, miR-205, miR-449, miR-216, miR-375, miR-429, miR-302b, miR-30a-5p, miR-30a-3p, miR-30b, miR-32, miR-345, miR-30	Upregulated	RT-qPCR	Bekris et al. (2013)
	miR-34a, miR-125b, miR-146a	Downregulated	RT-qPCR	Kiko et al. (2014)
	miR-154, miR-15b, miR-214, miR-10a, miR-99a, miR-10b, miR-181a, miR-497, miR-195, miR-125, miR-455, miR-221, miR-126, miR-328b, miR-146b, miR-199a, miR-451, miR-127, miR-194, miR-422, miR-195, miR-142-5p, miR-181c, miR-14	Downregulated	RT-qPCR	Bekris et al. (2013)
	miR-9, miR-101	Downregulated	Illumina TruSeq Small RNA sequencing	Burgos et al. (2014)
CSF exomes	miR-125b-5p	Upregulated	Microarray, RT-qPCR	McKeever et al. (2018)
	miR-331-5p, miR-29c, miR-485-5p, miR-136-3p, miR-132-5p, miR-16-2	Downregulated	TaqMan assays, RT-qPCR	Gui et al. (2015)
	miR-16-5p, miR-451a, miR-605-5p	Downregulated	Microarray, RT-qPCR	McKeever et al. (2018)
Brain	miR-9, miR-125b, miR-132, miR-128	Upregulated	Microarray, Northern Analysis	Lukiw (2007)
	miR-146a	Upregulated	Microarray, Northern Analysis	Lukiw et al. (2008)
	miR-146a	Upregulated	DNA Array	Cui et al. (2010)
	miR-9, miR-146a, miR-125b	Upregulated	Microarray, Northern Analysis	Sethi and Lukiw, (2009)
	miR-125, miR-26a, miR-423, miR-27b, miR-422a, miR-30e-5p, miR-381, miR-34a, miR-92, miR-145, miR-200c	Upregulated	RT-qPCR	Cogswell et al. (2008)
	miR-146a	Upregulated	Microarray, northern blot	Cui et al. (2010)	
	miR-107	Downregulated	Microarray, Northern Blotting	Wang et al. (2008, p. 1)
	miR-212, miR-9, miR-146b, miR-132	Downregulated	RT-qPCR	Cogswell et al. (2008)
	miR-298, miR-328	Downregulated	Northern Blotting	Boissonneault et al. (2009)
	miR-29a	Downregulated	Microarray, RT-qPCR	Shioya et al. (2010, p. 3)
	miR-29a-1, miR29b-1, miR-363, miR-181c, miR-106b, miR-15a, miR-101, miR-93, miR-9, miR-19b, miR-15a, miR-22, miR-26b, miR-210, let-7i	Downregulated	Microarray, northern blot, RT-qPCR	Hébert et al. (2008)
	miR-9, miR-132, miR-98, miR-212, miR-146b, miR-425, miR-30c	Downregulated	RT-qPCR	Cogswell et al. (2008)
	miR-106b	Downregulated	Northern Blotting, qPCR,	Wang et al. (2008)
	miR-15a	Downregulated	qPCR,	Hébert et al. (2010)
	miR-485-5p	Downregulated	qPCR,	Faghihi et al. (2010)
	miR-124	Downregulated	qPCR	Smith et al. (2011)
Cortex	miR-424	Upregulated	LNA-microarrays, northern blot analysis	Wang W.-X. et al. (2011)
	miR-137, miR-9, miR-29b, miR-29a, miR-181c,	Downregulated	RT-qPCR	Geekiyanage and Chan, (2011)
	miR-212	Downregulated	LNA-microarrays, northern blot analysis	Wang W.-X. et al. (2011)

### 4.2 MicroRNA biomarkers in Parkinson’s disease

Parkinson’s disease (PD) is a standard ND that affects about 1% of individuals over the age of 60 (Tysnes and Storstein, 2017). It is associated with the severe loss of substantia nigra par compacta dopaminergic (DA) neurons. Its pathophysiology includes both hereditary and environmental risk factors. The clinical symptoms encompass motor dysfunction, such as bradykinesia, resting tremors, and postural instability. Autonomic dysfunction, such as constipation or erectile dysfunction, and depression can occur even before the onset of motor dysfunctions. Late-onset disease begins with detecting genetic mutations in the α-synuclein (SNCA) and leucine-rich repeat kinase 2 (LRRK2) genes. Additionally, mutations were identified in the Parkin (PARK2), PTEN-induced putative kinase 1 (PINK1), and oncogene DJ1 (DJ1) genes, which are associated with an early onset of disease (Coppedè, 2012).

Parkinson’s disease is diagnosed primarily based on clinical symptoms and neurological examinations (Zhang W.T. et al., 2022). However, this ND remains challenging to diagnose and treat because its symptoms may differ from one patient to the next and may overlap with other related conditions, called atypical Parkinsonian disorders. This is due to several complicating factors, including gene mutation, neurotrophic factor insufficiency, oxidative stress, excitotoxicity, immunological dysregulation, mitochondrial malfunction, and apoptosis (Angelova, 2021; Tolosa et al., 2021). As a result of the heterogeneity of the disease, reliable laboratory diagnostic tests are still lacking.

There is a widely recognized need for more diagnostic tools for PD. However, imaging techniques such as MRI and DaTscan of the brain and the unified Parkinson’s Hoehn-Yahr scale have been successfully used in PD diagnosis. Novel biomarkers are also urgently needed to address the shortcomings of the current diagnostic approach (Zhang W.T. et al., 2022). Therefore, miRNAs may be valuable biomarkers for PD (Roser et al., 2018; Chen et al., 2021). For example, miR-4639-5p has a sensitivity of 94% and a specificity of 85% in diagnosing PD (Chen et al., 2017), while miR-494 exhibits low diagnostic efficiency, with 61% sensitivity and 79% specificity (Li et al., 2021). However, the joint use of miR-124 and miR-494 has shown diagnostic efficacy in distinguishing PD patients from healthy controls. This approach achieved a sensitivity of 86% and a specificity of 85% (Li et al., 2021). Table 2 presents a compilation of miRNAs that can be used to diagnose PD.

**Table 2:** List of reported miRNAs in Parkinson’s disease.

Source	miRNA	Expression	Methods	References
Serum	miR-29c	Upregulated	RT-qPCR	Ozdilek and Demircan (2021)
	miR-324-3p, miR-223-5p, miR-24	Upregulated	TaqMan assay	Vallelunga et al. (2014)
	miR-373, miR-30c-5p	Upregulated	RT-qPCR	Zhang et al. (2020)
	miR-16-2-3p, miR-30e-3p, miR-1294, miR-338-3p	Upregulated	TruSeq small RNA sequencing	Burgos et al. (2014)
	miR-24, miR-195	Upregulated	RT-qPCR	Cao et al. (2017)
	miR-195	Upregulated	Solexa sequencing followed by	Ding et al. (2016)
	miR-223-3p	Upregulated	RT-qPCR	Mancuso et al. (2019)
	miR-146a-5p, miR-132-3p	Downregulated	RT-qPCR	Shu et al. (2020)
	miR-29c, miR-29a	Downregulated	RT-qPCR	Bai et al. (2017)
	miR-19b, miR-29a, miR-29c	Downregulated	RT-qPCR	Botta-Orfila et al. (2014)
	miR-30c, miR-339-5p, miR-148b	Downregulated	TaqMan Assay	Vallelunga et al. (2014)
	miR-19b	Downregulated	RT-qPCR	Cao et al. (2017)
	miR-29c, miR-214, miR-146a, miR-221	Downregulated	RT-qPCR	Ma et al. (2016)
	miR-181, miR-185, miR-221, miR-15b	Downregulated	Solexa sequencing followed by qPCR	Ding et al. (2016)
	miR-193a-3p, miR-141, miR-146b-5p, miR-214	Downregulated	Solexa sequencing followed by RT-qPCR	Dong et al. (2016)
Plasma	miR-132	Upregulated	RT-qPCR	Yang et al. (2019b)
	miR-105-5p	Upregulated	RT-qPCR	Yang et al. (2019a)
	miR-4639-5p	Upregulated	Microarray, RT-qPCR	Chen et al. (2017)
	miR-27a	Upregulated	RT-qPCR	Chen et al. (2018)
	miR-125a-3p, miR-181c, miR-137, miR-331-5p, miR-454, miR-196b, miR-193a	Upregulated	TaqMan low-density arrays RT-qPCR	Cardo et al. (2013)
	miR-221-3p, miR-133b	Upregulated	RT-qPCR	Chen et al. (2021)
	miR-137	Upregulated	RT-qPCR	Li N. et al. (2017)
	miR-626, miR-450b-3p, miR-505, miR-1826	Downregulated	Microarray, RT-qPCR	Khoo et al. (2012)
	miR-124	Downregulated	RT-qPCR	Li N. et al. (2017)
	let-7a, miR-142-3, let-7f, miR-222	Downregulated	RT-qPCR	Chen et al. (2018)
	miR-124	Downregulated	RT-qPCR	Li N. et al. (2017)
Blood	miR-199b, miR-1274b, miR-4293, miR-20a, miR-671, miR-18b-3p, miR-150, miR-1249, miR-21, miR-378c	Upregulated	NGS, Microarray	Soreq et al. (2013)
	miR-16-1, miR-320a, miR-320c-1, miR-320b-1, miR-92b, miR-769	Downregulated	NGS, Microarray	Soreq et al. (2013)
Peripheral blood	miR-16-2, miR-26a, miR-30a	Upregulated	RT-qPCR	Margis et al. (2011)
	miR-29a, miR-22	Downregulated	RT-qPCR	Margis et al. (2011)
	miR-301a, miR-126-5p, miR-151-3p, miR-126-3p, miR-30c, miR-19b, miR-26a, miR29c, miR-29b, miR-199a-5p, miR-374b, miR-374a, miR-335, miR-199a-3p, miR-30b, miR-147, miR-28-5p, miR151-5p	Downregulated	Microarray, ChIP-seq, ChIP real-time PCR	Martins et al. (2011)
Cerebrospinal fluid	let-7f-5p	Upregulated	NGS	dos Santos et al. (2018)
	miR-205	Upregulated	RT-qPCR	Marques et al. (2017)
	miR-144-5p, miR-200a-3p, miR-542-3p	Upregulated	RT-qPCR	Mo et al. (2016)
	miR-24	Downregulated	RT-qPCR	Marques et al. (2017)
	miR-626	Downregulated	RT-qPCR	Qin et al. (2021)
	miR-423-5p, miR-27a-3p	Downregulated	RT-qPCR	dos Santos et al. (2018)
CSF exomes	let-7 g-3p, miR-153, miR-10a-5p, miR-409-3p	Upregulated	TaqMan low-density array, RT-qPCR	Gui et al. (2015)
	miR-1, miR-19b-3p	Downregulated	TaqMan low-density array	Gui et al. (2015)
Saliva	miR-145-3p, miR-874	Upregulated	RT-qPCR	Chen Y. et al. (2020)
	miR-132-3p	Upregulated	RT-qPCR	Gong et al. (2022)
	miR-133b	Downregulated	Northern blot analysis, RT-qPCR, luciferase assay	Kim et al. (2007)
			
	miR-223, miR-153	Downregulated	RT-qPCR	Cressatti et al. (2020)
	miR-34c, miR-874	Downregulated	Microarray, qPCR	Miñones-Moyano et al. (2011)
Substantia nigra pars compacta and amygdala	miR-26b, miR-21, miR-3736, miR-106a, miR-301b, miR-224	Upregulated	RT-qPCR	Alvarez-Erviti et al. (2013)
	miR-198, miR-548d, miR-485-5p, miR-135b	Downregulated	TaqMan assay	Cardo et al. (2014)

Mouse models for PD have shown miR-124’s potential neuroprotective properties (Zhang F. et al., 2022). Parkinson’s disease mice treated with miR-124 showed improved motor deficits, reduced dopaminergic neuron loss, and reduced oxidative stress. It also targets Axin 1 (the protein encoded by the AXIN1 gene) and stimulates the Wnt/β-catenin signaling pathway, suppressing PD progression. Results indicate that miR-124 may be an effective treatment for PD (Kanagaraj et al., 2014; Zhang F. et al., 2022). Studies should focus on identifying strategies to increase miR-124 delivery to the brain and assessing the safety of miR-124 treatment in human subjects. Therefore, further research is needed to explore miR-124’s therapeutic potential.

### 4.3 MicroRNA biomarkers in amyotrophic lateral sclerosis

Amyotrophic lateral sclerosis is an incurable disease that gradually causes the loss of motor neurons in the brain and spinal cord (De Felice et al., 2012; Mead et al., 2023). Motor neurons are the only cells that degenerate and die in ALS, but there is evidence that a single cell type does not cause the disease. Non-neuronal cells in the environment, including microglia, astrocytes, muscle, and T cells, play a critical role in disease development (Vahsen et al., 2021)—motor neuron degeneration results in progressive weakening of the limbs, respiratory muscles, and bulbar muscles.

In the early stages of the disease, symptoms can vary depending on the extent of damage to motor neurons in the brain and spinal cord. Hyperreflexia and muscle cramps are upper motor neuron (UMN) injury symptoms. Lower motor neuron failure, on the other hand, results in generalized weakness, fasciculation, muscular atrophy, muscle cramps, and hyporeflexia (Brown and Al-Chalabi, 2017). Patients with ALS often have difficulty eating or swallowing and have slurred or nasal speech. About 25% of ALS patients demonstrate bulbar involvement, which is less common than limb involvement. Most patients have lower motor neuron (LMN) and UMN symptoms during the condition, caused by damage in the spinal and brainstem regions. Patients with ALS have a poor prognosis, and death generally occurs 2–4 years after the onset of symptoms owing to bulbar dysfunction and respiratory failure. However, in rare cases, patients with ALS may have a longer life expectancy (Phukan et al., 2007; Brown and Al-Chalabi, 2017; Grad et al., 2017; Ricci et al., 2018; Masrori and Van Damme, 2020; Zakharova and Abramova, 2021). Currently, there is no laboratory test available to diagnose ALS definitively. The diagnosis of ALS is typically established through clinical examinations, laboratory tests, and the exclusion of other conditions. The process of diagnosing ALS can be challenging, and it often involves a team of healthcare professionals, including neurologists. It is difficult to provide timely and effective treatment because it takes around a year from the initial symptoms to a diagnosis (Ricci et al., 2018; Štětkářová and Ehler, 2021).

Studies have proposed miRNAs as diagnostic marker candidates showing variable expression rates in ALS patients. It indicates that these miRNAs may regulate the development of diseases (De Felice et al., 2012; Freischmidt et al., 2013; Feneberg et al., 2014). The lists of miRNAs reported in ALS are mentioned in Table 3. For example, miR-155, a pro-inflammatory miRNA, is upregulated in spinal cord animal models and ALS patients. Interestingly, its inhibition has improved survival and motor function in experimental animals (Butovsky et al., 2012; Yu et al., 2013). Skeletal muscle exhibits elevated levels of miR-206 expression, known to regulate muscle development. It is downregulated in ALS patients and animal models, and its overexpression improves motor function in animal models (Williams et al., 2009). Other ALS-related miRNAs are miR-9, which regulates astrocyte function, and miR-124, which regulates neuronal differentiation and neurogenesis (Hawley et al., 2019; Zhao et al., 2021; Martinez and Peplow, 2022). Additionally, the miR-21, miR-146a, miR-132, and miR-124 have been implicated in the pathogenesis of ALS (Kovanda et al., 2018; Zhou F. et al., 2018; Barbosa et al., 2021; Bai and Bian, 2022; Gomes et al., 2022). Researchers have found that these miRNAs can change neuroinflammatory responses, suggesting they could be therapeutic targets (Ravnik-Glavač and Glavač, 2020; Martinez and Peplow, 2022). A study showed that miR-338-3p modulates glutamate transporter EAAT2 expression and that its downregulation may contribute to ALS (Rosenblum and Trotti, 2017; Sun et al., 2018; Tian et al., 2021).

**Table 3:** List of reported microRNAs in amyotrophic lateral sclerosis.

Source	miRNA	Expression	Methods	References
Serum	miR-338-3p	Upregulated	RT-qPCR	De Felice et al. (2014)
	miR-106b, miR-206	Upregulated	Microarray, qPCR	Toivonen et al. (2014)
	miR-192-3p, miR-133b, miR-19a-3p, miR-144-5p, miR-195-5p, miR-133a-3p	Upregulated	RT-qPCR	Raheja et al. (2018)
	miR-206, miR-133b, miR-133a	Upregulated	RT-qPCR	Tasca et al. (2016)
	miR-143-3p, iR-206,	Upregulated	RT-qPCR	Waller et al. (2017)
	miR-142-3p	Upregulated	Microarray, RT-qPCR	Matamala et al. (2018)
	miR-1249-3p	Downregulated	Microarray, RT-qPCR	Matamala et al. (2018)
	miR-320b, miR-320c, let-7d-3p, miR-320a, miR-425-5p, miR-139-5p	Downregulated	RT-qPCR	Raheja et al. (2018)
	miR-146a, miR-27a, miR-149,	Downregulated	RT-qPCR	Tasca et al. (2016)
	let-7b, miR-143-3p, miR-143-5p, miR-132-5p, miR-132-3p,	Downregulated	RT-qPCR	Freischmidt et al. (2013)
	miR-374b-5p	Downregulated	RT-qPCR	Waller et al. (2017)
	miR-3665, miR-4745-5p, miR-4530, miR-1915-3p	Downregulated	Microarray, RT-qPCR	Freischmidt et al. (2014)
	miR-1234-3p, miR-1825	Downregulated	Microarray, RT-qPCR	Freischmidt et al. (2015)
Serum exomes	miR-27a-3p	Downregulated	RT-qPCR	Xu et al. (2018)
Plasma	miR-206, miR-424	Upregulated	Microarray, RT-qPCR	de Andrade et al. (2016)
	miR-4258, miR-663b, miR-4649-5p	Upregulated	Microarray, RT-qPCR	Takahashi et al. (2015)
	miR-26b-5p, miR-4299, let-7f-5p,	Downregulated	Microarray, RT-qPCR	Takahashi et al. (2015)
	miR-9, miR-335-5p, miR-129-3p	Downregulated	RT-qPCR	Sheinerman et al. (2017)
Whole blood	let-7b, miR-9, miR-338, miR-206, miR-132, miR-451a, miR-638, miR-663a, miR-124a	Upregulated	RT-qPCR	Vrabec et al. (2018)
	miR-338-3p	Upregulated	Microarray, RT-qPCR	De Felice et al. (2012)
	miR-193b, miR-3935, miR-451, miR-183	Downregulated	Microarray, RT-qPCR	Chen Y. et al. (2016)
	miR-451, miR-1275, miR-665, miR-638, miR-149, miR-328	Downregulated	Microarray, RT-qPCR	De Felice et al. (2012)
Peripheral blood	miR-584-5p, miR-93-5p, miR-550a-3p, miR-532-5p, miR-451a, miR-425-5p, miR-3p, miR-30c-5p, miR-30b-5p, miR-28-3p, miR-27b-3p, miR-26b-5p, miR-26a-5p, miR-23a-3p, miR-223-3p, miR-221-3p, miR-22-3p, miR-186-5p, miR-183-5p, miR-182-5p, miR-16-5p, miR-151b, miR-151a-5p, miR-15b-5p, miR-15a-5p, miR-148b-3p, miR-148a-3p, miR-144-5p, miR-130b-3p, miR-130a-3p, miR-128-3p, miR-106b-3p, miR-103a-3p, let-7i-5p, let-7 g-5p, let-7f-5p, let-7d-5p, let-7a-5p.	Downregulated	NGS, RT-qPCR	Liguori et al. (2018)
CSF	miR-338-3p	Upregulated	RT-qPCR	De Felice et al. (2014)
	miR-9-5p, miR-127-3p, miR-143-3p, miR-27b-3p, miR-124-3p, miR-125b-3p	Upregulated	Illumina Small RNA Sequencing, RT-qPCR	Waller et al. (2018)
	miR-181a-5p	Upregulated	RT-qPCR	Benigni et al. (2016)
	miR-574-5p, miR-143-5p,	Upregulated	RT-qPCR	Freischmidt et al. (2013)
	miR-124	Downregulated	RT-qPCR	Yelick et al. (2020)
	miR-143-3p, miR-132-5p, miR-132-3p,	Downregulated	RT-qPCR	Freischmidt et al. (2013)
	let7f-5p, let7a-5p, let7b-5p, miR-21-5p, miR-148-3p, miR-195a-5p, miR-15b-5p	Downregulated	RT-qPCR	Benigni et al. (2016)
	let7f-5p, miR-142-5p, miR-28-3p, miR-150-5p, miR-16-5p, miR-92a-5p, miR-146a-3p, miR-378a-3p	Downregulated	NGS	Waller et al. (2018)

In 2021, Dobrowolny and colleagues used absolute RT-qPCR quantification and next-generation sequencing to examine the levels of miRNA circulating in healthy individuals and ALS patients’ blood. They discovered a correlation between miR-151a-5p, miR-133a, and miR-206 levels in ALS patients and reduced function loss. Therefore, these miRNAs may help predict a person’s future performance. It is observed that miR-206 and miR-151a-5p expressions are elevated in the early stages of ALS, but expression declines in the subsequent moderate and severe stages (Dobrowolny et al., 2021). However, miR-199a-5p and miR-133a remained low throughout the disease. The findings suggest that miR-133a has diagnostic significance in the severe and moderate stages, whereas miR-199a-5p is essential in the early and terminal stages (Dobrowolny et al., 2021). Clinical researchers may be able to tailor treatment strategies based on miRNAs as prognostic indicators for ALS. Additionally, several miRNAs associated with disease severity and progression could be targeted to treat ALS (Dobrowolny et al., 2021).

In conclusion, studying the roles and functions of differentially expressed miRNAs in ALS might offer essential insights into the disease’s underlying processes and potential treatment targets. Therefore, miRNAs should be further investigated in ALS research.

### 4.4 MicroRNA biomarkers in Huntington’s disease

In 1872, the American physician George Huntington published the first clinical description of HD. This incurable autosomal dominant genetic disorder results in neuronal degeneration due to repeated replication of the cytosine-adenine-guanine (CAG) trinucleotide in the Huntingtin gene. Symptoms of HD typically appear between 39 ± 5 years of age, although they can appear in children (Jacobsen et al., 2010). They include uncontrollable excessive motor movements and cognitive or behavioral symptoms, generally discovered after the disease progresses. The unified HD rating, which provides an overall rating system based on motor, behavioral, mental, and functional assessments, can measure disease progression. In imaging studies, such as MRI or computed tomography (CT), the caudate nuclei may show evidence of atrophy early in the disease (Roos, 2010; Finkbeiner, 2011; Ghosh and Tabrizi, 2018).

Huntingtin gene was discovered on chromosome 4p16.3 in 1993 (MacDonald et al., 1993). The gene integrity is compromised by the repeated irregular expansion of the CAG triplet in the gene’s first exon. Normal brain development and function depend on translating the translated protein corresponding to the Htt allele, which consists of 6–35 CAG triplet repeats. In contrast, a CAG triplet expansion of 40 or more repeats indicates complete penetration, which indicates an abnormality. Alleles with 36–39 CAG repeats have low penetration. In contrast, CAG repeats ranging from 27 to 35 fall within the approved range and are known as intermediate or unstable alleles due to their ability to change size during reproduction (Southwell et al., 2015; Chen and Wolynes, 2017; Jarosińska and Rüdiger, 2021).

The mutant Huntingtin protein (mHTT) is produced by a mutation in the Htt gene due to a tremendous polyglutamine repeat that aggregates in the brain and forms toxic clumps (Coppen and Roos, 2017; Fodale et al., 2020). An estimated frequency of 5–10 per 100,000 of neurodegeneration results from mobility problems, cognitive impairment, and mental symptoms (Mestre et al., 2009; Roos, 2010). The mHTT protein can exist in monomeric and aggregated forms, and research has focused on developing antibodies against both forms for diagnostic and therapeutic purposes. Evidence shows that the soluble monomeric huntingtin protein and the aggregated huntingtin protein may also be toxic to neurons. A mouse model of HD demonstrated that antibodies against monomeric huntingtin protein prevented neuronal death (Southwell et al., 2015; Fodale et al., 2020; Tabrizi et al., 2020; Denis et al., 2023). According to these findings, HD can be treated by targeting the monomeric huntingtin protein. Nevertheless, more research is needed to understand better the roles of monomeric and aggregated huntingtin proteins in HD and develop effective treatments to treat these forms (Southwell et al., 2015). Further research is required to determine whether targeting monomeric huntingtin proteins can be a proper therapeutic strategy for HD.

The clinical symptoms of HD can be classified into three types: motor symptoms, mental challenges, and cognitive impairment (Tabrizi et al., 2020). The disease causes involuntary movements, such as chorea. There are several dyskinesias associated with Parkinson’s syndrome (Heo and Scott, 2017), including chorea-like symptoms, ataxia, dystonia, and Parkinson’s syndrome. Psychological symptoms such as anxiety and anger may occur before dyskinesia occurs in HD patients (Goh et al., 2018). Further, executive dysfunction is primarily responsible for HD patients’ cognitive impairment. According to some studies (Bayliss et al., 2019), cognitive impairment could worsen as CAGs multiply. MiRNAs regulate gene expression and contribute to HD pathogenesis. The dysregulation of miR-9 in individuals affected by HD has been associated with neuronal loss (Packer et al., 2008). MiR-124a controls neuronal differentiation and improves HD motor symptoms in mouse models (Lee et al., 2017; Martinez and Peplow, 2021). A list of miRNA biomarkers reported in the literature is mentioned in Table 4. These miRNA biomarkers can provide insight into the molecular mechanisms underlying HD and can be used to design therapeutic strategies for HD. As such, miRNAs are essential for understanding and managing HD.

**Table 4:** List of reported miRNAs in Huntington’s disease.

Source	MiRNA	Expression	Method	References
Plasma	miR-34b	Upregulated	RT-qPCR, Locked nucleic acid (LNA) Microarray	Gaughwin et al. (2011)
	miR-128, miR-338-3p, miR-425-5p, miR-628-3p, miR-30d-5p, miR-22-5p, miR361-5p, miR-223-3p, miR-942, miR-877-5p, miR-222-3p, miR-223-5p, miR-130b-3p, miR-130b-3p	Upregulated	RT-qPCR	Díez-Planelles et al. (2016)
	miR-486-5p, miR-10b-5p,	Upregulated	RT-qPCR	Hoss et al. (2015b)
Peripheral leukocytes	miR-9	Downregulated	RT-qPCR	Chang et al. (2017)
CSF	miR-4317, miR-140-5p, miR-135b-3p, miR-8082, miR-3928-5p, miR-520f-3p	Upregulated	HTG EdgeSeq system	Reed et al. (2018)
Cortex	miR-151-3p, miR-451, miR-92a, miR-100, miR-27b, miR-219-2-3p, miR-16	Upregulated	RNAseq, Microarray RT-qPCR	Martí et al. (2010)
	miR-330, miR-29a	Upregulated	RT-qPCR	Johnson et al. (2008)
	miR-196b-5p, miR-10b-5p, miR-12475p, miR-196a-5p, miR-615-3p	Upregulated	Illumina miRNA sequencing (miRNA-seq)	Hoss et al. (2014)
	miR-10b-5p, miR-28-5p, miR-218-1-3p, miR-615-3p, miR-1535p, miR-10b-3p, miR-302a-3p, miR-320b, miR-144-3p, miR-223-3p, miR-302a-5p, miR-126-5p, miR-1445p, miR-200c-3p, miR-29a-3p, miR-486-3p, miR-888-5p, miR-5695, miR-548, miR-148a-5p, miR-iR-891a-5p, miR-16-2-3p, miR-208b-3p, miR-363-3p, miR-148a-3p, miR-199a-5p, miR-150-5p, miR-486-5p, miR-106a-5p, miR142-5p, miR-549a, miR-141-3p, miR-3065-5p, miR-224-5p, miR-452-5p, miR-4443, miR-101-5p, miR-483-5p, miR-2114-5p, miR-1945p, miR-196a-5p, miR-196b-5p, miR-214-5p, miR-1247-5p	Upregulated	Illumina’s TruSeq Small RNA	Hoss et al. (2015a)
	miR-483-3p, miR-139-3p, miR-433, miR-181d, miR-382, miR-222	Downregulated	RNAseq, Microarrays, RT-qPCR	Martí et al. (2010)
	miR-9, miR-29b, miR-124	Downregulated	Microarray, RT-qPCR	Packer et al. (2008)
	miR-132	Downregulated	RT-qPCR	Johnson and Buckley (2009)
	miR-3200-3p, miR-4516, miR-138-2-3p, miR-431-5p, miR-132-3p, miR-23b-5p, miR-448, miR-34b-3p, miR-1538, miR-490-5p, miR-1224-5p, miR-1298-5p, miR-1298-3p, miR-346, miR-663a, miR-4449, miR-5680, miR-1298-3p, miR-4787-3p, miR-129-1-3p, miR-1185-1-3p, miR-670-3p, miR-129-5p, miR-135b-5p, miR-4488, miR-34c-5p, miR-1264, miR-433-3p, miR-219b-3p, miR-663b, miR-181a3p, miR-877-5p, miR-3139, miR-6734-5p	Downregulated	Illumina’s TruSeq Small RNA	Hoss et al. (2015a)
STHdh cells	miR-146a	Downregulated	RT-qPCR	Das et al. (2015), Sinha et al. (2010, 2011)

### 4.5 MicroRNA biomarkers in multiple sclerosis

Multiple sclerosis is an autoimmune disease impacting the brain and spinal cord, resulting in chronic neurological impairment. A significant degree of physical disability results from demyelinating lesions that damage myelin and the axons of the CNS (Arneth and Kraus, 2022). Although the exact cause of MS is unknown, it is likely to be influenced by genetic and non-genetic factors, such as viral infections. Axons and myelin are permanently destroyed by an immune-mediated inflammatory process (Lawrence Steinman, 1996; O’Gorman et al., 2012; de Faria et al., 2013; Dobson and Giovannoni, 2019; Gao et al., 2021; Arneth and Kraus, 2022; Lamb, 2022).

Multiple sclerosis pathophysiology involves the formation of localized demyelination areas or plaques caused by the destruction of oligodendroglia cells that produce myelin (a fatty substance that protects nerve fibers and insulates them). MS pathophysiology is also marked by perivascular inflammation, further damaging the oligodendroglia cells and surrounding tissues. MS is classified as an autoimmune disease involving T-cells, where the immune system mistakenly attacks its healthy cells, including those in the CNS. MS can be divided into two main phases: relapsing–remitting and secondary progressive. The first phase is characterized by episodes of inflammation followed by periods of relative calm or remission, often affecting younger patients. The second phase, known as primary progressive MS, is marked by a steady deterioration of neurological function from the onset of the disease without distinct relapses or remissions. Secondary progressive MS occurs when there is progression after an initial period of relapsing–remitting disease (Lamb, 2022). MS is also characterized by perivenular infiltration of lymphocytes and macrophages. The prevalence of MS is rising globally, notably more prevalent in countries with temperate climates. Risk factors for MS include age, sex, race, vitamin D levels, smoking, childhood obesity, exposure to ultraviolet radiation (UVR), autoimmune diseases, genetic background, and Epstein–Barr virus (EBV) infection. Individuals with Northern European ancestry have a higher susceptibility to MS, while those of Asian, African, or Native American heritage have the least susceptibility (O’Gorman et al., 2012; Dobson and Giovannoni, 2019; Chacko et al., 2021).

Several studies have identified specific miRNAs that are differentially expressed in MS patients compared to healthy individuals, and some of these miRNAs have been associated with disease subtypes and clinical parameters. Specific miRNAs were associated with MRI-based phenotypes, suggesting a link to blood–brain barrier pathology (Pietrasik et al., 2021; Lamb, 2022). Table 5 presents a selection of miRNAs discovered in different body fluids. For instance, one study that involved four observational cohorts found that miR-484, miR-320a, miR-486-5p, miR-320c, and miR-140-5p expression is significantly different in MS patients than in healthy people (Regev et al., 2018). One study found that miR-92a-1 levels differed between relapsing–remitting MS (RRMS) patients and healthy controls and secondary progressive MS (SPMS) patients. Furthermore, this miRNA was associated with disease duration and the Expanded Disability Status Scale (EDSS) (Pietrasik et al., 2021).

**Table 5:** List of reported miRNAs in multiple sclerosis.

Source	MiRNA	Expression	Method	References
Serum	miR-223, miR148b, miR-24, miR-34b, miR-324-3p	Upregulated	TaqMan low-density arrays	Vallelunga et al. (2014)
	miR-15b, miR-23a, miR-223	Downregulated	TaqMan, RT-qPCR	Fenoglio et al. (2013)
	miR-572	Downregulated	RT-qPCR	Mancuso et al. (2015)
	miR-339-5p	Downregulated	TaqMan low-density arrays,	Vallelunga et al. (2014)
	miR-15a-3p, miR-34c-5p	Downregulated	Microarray, RT-qPCR	Perdaens et al. (2020)
Plasma	miR-1826, miR-614, miR-422a, miR-572, miR-648, miR-22	Upregulated	Microarray Analysis	Siegel et al. (2012)
	let-7d, miR-140-3p, miR-22, miR-30e, miR-574-3p, miR-210, miR-92a-1, miR-145, miR-500	Upregulated	RT-qPCR	Gandhi et al. (2013)
	miR-155	Upregulated	RT-qPCR	Thamilarasan et al. (2012)
	miR-1979	Downregulated	Microarray, RT-qPCR	Siegel et al. (2012)
Blood	miR-18b, miR-599, miR-96	Upregulated	RT-qPCR	Otaegui et al. (2009)
	miR-223, miR-145, miR-584, miR-491-5p, miR-186, miR-664, miR-422a, miR-1275, miR-142-3p	Upregulated	Microarray	Keller et al. (2009)
	miR-16-2-3p	Upregulated	NGS, microarray analysis, RT-qPCR	Keller et al. (2014)
	miR-150-5p, miR-155-5p	Upregulated	Microarray, RT-qPCR	Perdaens et al. (2020)
	miR-7-1-3p, miR-20a-5p	Downregulated	NGS, microarray analysis, RT-qPCR	Keller et al. (2014)
	miR-15a-3p, miR-34c-5p	Downregulated	Microarray, RT-qPCR	Perdaens et al. (2020)
	miR-124	Downregulated	RT-qPCR	Amoruso et al. (2020)
Peripheral blood	miR-146b, miR-21, miR-326, miR-142-3p, miR-155, miR-146a	Upregulated	RT-qPCR	Ma et al. (2014)
	miR-125a, miR-200c, miR-146b	Upregulated	Microarray analysis, RT-qPCR	Yang et al. (2014)
	miR-145	Upregulated	RT-qPCR	Søndergaard et al. (2013)
	miR-152	Downregulated	Microarray analysis, RT-qPCR	Yang et al. (2014)
	miR-15a, miR-15b, miR-328, miR-181c	Downregulated	RT-qPCR	Ma et al. (2014)
	miR-17, miR-20a	Downregulated	Microarray analysis, RT-qPCR	Cox et al. (2010)
Cerebrospinal fluid	miR-181c, miR-633	Upregulated	RT-qPCR	Haghikia et al. (2012)
	miR-15a-3p, miR-34c-5p	Downregulated	Microarray, RT-qPCR	Perdaens et al. (2020)
	miR-922	Downregulated	RT-qPCR	Haghikia et al. (2012)

A study by Helmond and colleagues analyzed an extensive group of MS patients using brain imaging (Gandhi et al., 2013). MicroRNAs were also associated with the EDSS and the duration of the disease (Gandhi et al., 2013). MicroRNAs were associated with various MRI-based phenotypes, indicating a possible relationship to the blood–brain barrier. The most reliable indicators of MRI subgroups were miR-22-3p, miR-345-5p, and miR-361-5p (Hemond et al., 2019).

MicroRNAs dysregulated expression in MS and association with disease parameters make them promising candidates for further investigation and development as MS biomarkers. Researchers have found that miRNAs can be used to distinguish between different types of MS and monitor their progression. Moreover, they emphasize the need for ongoing research to validate and further explore miRNAs as clinical biomarkers for MS. As such, miRNAs can be handy tools for diagnosing and treating the disease.

## 5 Role of microRNA in post translational modification of neurodegenerative diseases

### 5.1 Alzheimer disease

In the complex network of AD pathology, miRNAs function as regulatory elements within the post-translational modification (PTM), orchestrating molecular events that contribute to the onset and progression of the disease. These PTMs encompass a diverse array of processes, including acetylation, carbamylation, glycation, methylation, nitration, sumoylation, truncation, ubiquitination, and phosphorylation. Through the regulation of these pathways, miRNAs assume crucial positions in molding the molecular processes driving AD development and etiology (Singh et al., 2023). The miRNAs can influence tau protein expression in AD through multiple mechanisms like phosphorylation, splicing and acetylation. Tables 6, 7 scope key miRNAs involved in ADs pathology through their regulation of PTMs, driving disease progression and onset (Banzhaf-Strathmann et al., 2014; Salta et al., 2016; Sharma and Lu, 2018; Silvestro et al., 2019; Praticò, 2020; Lauretti et al., 2021; Park and Moon, 2022).

**Table 6:** Role of microRNA in tau protein metabolism.

MicroRNA	Abnormal tau protein function	References
miR-219	Targets the MAPT (microtubule-associated protein tau) gene, influencing tau protein production.	Santa-Maria et al. (2015)
miR-106b	Restriction of Aβ42 phosphorylated tau through its specific targeting of the Fyn gene (a proto-oncogene)	Liu et al. (2016)
miR-124-3p	Suppresses the process of converting CAPN1 mRNA into protein, hence decreasing the occurrence of aberrant tau phosphorylation.	Zhou et al. (2019)
miR-219-5p	The activity of GSK3 is reduced, which leads to the inhibition of tau phosphorylation.	Li et al. (2019a)
miR-146a	Causes excessive phosphorylation of tau via altering the ROCK1 (Rho-associated, coiled-coil containing protein kinase 1)/PTEN signaling pathway.	Wang G. et al. (2016)
miR-92a	It is upregulated, causing anxiety via the miR-92a/vesicular GABA transporter (vGAT)/γ-aminobutyric acid (GABA) signaling pathway.	Li X. et al. (2017)
miR-137	Inhibits the phosphorylation of tau through the suppression of CACNA1C gene expression.	Jiang et al. (2018)
miR-138	Targets the RARA (Retinoic acid receptor α) /GSK3β pathway to increase tau phosphorylation.	Wang et al. (2015)

**Table 7:** Role of miRNA in Aβ metabolism.

MicroRNA	Role in Aβ metabolism	References
miR-339-5p	An up-regulation of BACE1 results from a decrease in miR-339-5p, which promotes the accumulation of Aβ.	Long et al. (2014)
miR-29c and miR-135b	It exerts a negative regulatory influence on the expression of BACE1 and has neuroprotective properties.	Zong et al. (2011) and Zhang et al. (2016, p. 1)
miR-15b	A component of Aβ metabolism that regulates the activity of BACE1.	Gong et al. (2017) and Li and Wang (2018)
miR-195	Participates in Aβ metabolism by regulating BACE1 activity	Zhang X. et al. (2018), Su et al. (2021), and Seyedaghamiri et al. (2023)
miR-124	Participates in Aβ metabolism via regulation of BACE1 activity.	Fang et al. (2012), Zhang et al. (2017)
miR-15/107	Downregulated in AD hippocampi, enhancing Aβ generation and phosphorylation of APP.	Moncini et al. (2017) and Li et al. (2019b)
miR-221	Expression levels are reduced in Alzheimer’s disease. Enhances the abundance of ADAM10, a constituent of α-secretases that plays a role in the breakdown of APP.	Manzine et al. (2018)
miR-151	Downregulates APH1 (Anterior pharynx defective-1), a member of the γ-secretase complex.	Xu et al. (2019)
miR-151	Downregulates APH1 (Anterior pharynx defective-1), a member of the γ-secretase complex.	Xu et al. (2019)
miR-107	Downregulated AD BACE1 and ADAM10 are targeted, influencing APP metabolism.	Nelson and Wang (2010), Augustin et al. (2012), and Goodall et al. (2013)
miR-140-5p	Increased expression in Alzheimer’s disease. Exerts a negative regulatory effect on ADAM10 and is triggered by Aβ.	Akhter et al. (2018)
miR-132	Downregulation in AD upregulates ITPKB (Inositol 1, 4, 5-trisphosphate 3-kinase B) and BACE1 activity.	Salta et al. (2016) and Zhu et al. (2016)
miR-330	VAV1 (Regulator of the small Rho-GTPase) is negatively regulated via the MAPK pathway, increasing Aβ generation	Zhou Y. et al. (2018)
miR-153	Suppresses the expression of APP	Long et al. (2012)
miR-200b/c	PTEN inhibition promotes Akt activation and mitigates the neurotoxicity induced by Aβ.	Wu Q. et al. (2016)
miR-302	Stimulating Akt via the PI3K (Phosphoinositide 3-kinases)/mTOR pathway mitigates the neurotoxicity induced by Aβ.	Li H.-H. et al. (2016)
miR-33	ATP-binding cassette transporter A1 (ABCA1) levels are decreased when miR-33 is upregulated in AD; this enzyme regulates Apolipoprotein E (APOE) lipidation and Aβ metabolism, thereby increasing Aβ levels.	Kim et al. (2015)
miR-188-5p	Downregulated in response to Aβ42 oligomerization in neurons of the hippocampus.	Lee et al. (2016)
miR-128	Targets peroxisome proliferator-activated receptor gamma (PPARG), a protein that enhances the development of Aβ pathology.	Huang et al. (2018)

Carbamylation involves the covalent addition of carbamoyl groups to lysine residues, especially prevalent in lysine-rich proteins like tau, which is associated with AD (Guru Krishna Kumar et al., 2018). As a result, it accelerates the formation of Aβ in AD and contributes to the neurodegenerative process. A variety of miRNAs have been implicated in carbamylation effects through their interactions with histone deacetylases (HDACs), including miR-34c (Zovoilis et al., 2011), miR-134 (Gao et al., 2010), miR-206 (Williams et al., 2009), and miR-124 (Johnson et al., 2008). Dysregulation of miRNA (miRNA)-histone deacetylase (HDAC) networks has been implicated in the pathogenesis of various neurological disorders. This suggests their potential as therapeutic targets of neurodegenerative diseases.

In the complex interplay between Alzheimer’s and diabetes, individuals with diabetes are at an increased risk for AD, driven by processes such as glycation and the involvement of advanced glycation end-products (AGEs) (Dukic-Stefanovic et al., 2001). Protein structure is disrupted by glycation, leading to amyloid aggregation in AD. These proteins are affected, significantly contributing to the pathology of the disease (Ott et al., 1999; Xu et al., 2009). A study reported, that miR-142 affects neuroinflammation and AD risk by targeting the receptor for advanced glycation end product (RAGE) pathway (Zhang R. et al., 2018).

MicroRNAs play key roles in methylation processes, influencing DNA methylation patterns, as well as the activity of methyltransferases in epigenetic regulation (Shi et al., 2004). Studies found that miR-let-7a-3 methylation (Brueckner et al., 2007), and miR-125b upregulation (Pogue et al., 2010) are linked to AD pathology, emphasizing the intrinsic relationship between miRNAs and methylation. This suggests that miRNAs are important mediators of epigenetic modifications in AD pathogenesis.

Nitration occurs when a nitrogen species, nitryl, is added to a protein to cause the protein to misfold, which leads to oxidative stress, misfolded proteins, oxidative stress and cellular damage (Radi, 2013). It is regulated by miRNAs such as miR-132, miR-212, and miR-188. These miRNAs affect neuronal viability and synaptic function in AD by modulating the production of neuronal nitric oxide synthase (NOS1) and reducing Aβ-induced toxicity through inhibition of NOS1 expression (Wang et al., 2017; Chen M. et al., 2020).

Sumoylation, involving the non-covalent attachment of SUMO (Small Ubiquitin-like Modifier) proteins to target proteins, modifies amyloid beta (Aβ) production and tau protein function in AD (Li et al., 2003; Wilkinson and Henley, 2010). It regulates key proteins such as APP intracellular domain (AICD), APP, impacting Aβ levels and plaque formation. In AD, tau sumoylation plays a role in tau phosphorylation and degradation (Tao et al., 2017; Liu et al., 2021). Prenatal exposure to specific substances may disrupt sumoylation processes in a way that could contribute to Alzheimer’s disease. Linked to this condition are alterations in miRNA expression, such as increased miR-489 and decreased levels of miR-33, miR-19b, and miR-509 (Wnuk et al., 2019).

Truncation occurs when proteins are shortened by specific proteases or by mutations that end mRNA translation prematurely by cleaving at specific sites in the protein (Wirths and Zampar, 2019). MicroRNAs play an important regulatory role in the expression and processing of the APP, which is central to the pathogenesis of AD. Several miRNAs have been found to modulate the activity of enzymes involved in APP processing, such as BACE1, calpain, and caspases, thereby influencing the production of Aβ peptides. For example, miR-16, miR-338-5p, miR-485-5p, miR-107, and miR-186 play a critical role in controlling BACE-1 expression, which affects Aβ production in AD (Maoz et al., 2017; Zhao et al., 2017; Wang et al., 2019; Kou et al., 2020). Specific miRNAs, such as miR-298, have been shown to reduce the levels of APP, BACE1, and Aβ peptides (Aβ40 and Aβ42) in cell culture models (Chopra et al., 2021) Several studies suggest that miR-132 reduces truncated tau fragments by targeting Calpain 2 and Caspase 3 (El Fatimy et al., 2018). Other miRNAs such as miR-195, miR-135a, miR-135b, and miR-339–5p also modulate BACE-1 expression, implicating their involvement in AD pathology (De Strooper and Karran, 2016). While miRNAs are involved in modulating APP levels and the production of Aβ peptides, their direct role in amyloid APP truncation requires further investigation to establish a conclusive link.

Ubiquitination, a PTM essential for protein degradation and cellular proteostasis, is tightly regulated by miRNAs like miR-7, miR-9, miR-181c, and ciRS-7. These miRNAs target E3 ubiquitin ligases and deubiquitinases involved in the ubiquitin-proteasome system, influencing misfolded protein clearance and toxic aggregate formation in AD (Schonrock et al., 2010; Zhao et al., 2016; Shi et al., 2017; Cochran et al., 2020).

Acetylation of lysine residues in tau and Aβ peptides has been linked to the pathogenic accumulation of tau and Aβ aggregates, contributing to synaptic dysfunction (Mattson, 2010; Cohen T.J. et al., 2011; Dodson et al., 2013). MicroRNAs such as miR-9, miR-212, and miR-181c have been identified by researchers to modulate the regulation of SIRT1. This leads to changes in tau acetylation via deacetylase activity (Schonrock et al., 2012; Weinberg et al., 2015).

Phosphorylation, a decisive PTM in signal transduction and cellular regulation, is tightly regulated by miRNAs such as the miR-29 family members, miR-16, miR-338-5p, miR-107, and miR-186 (Che et al., 2014; Kim et al., 2016). These miRNAs modulate the expression of protein kinases and phosphatases implicated in tau hyperphosphorylation and Aβ production. MiRBase reports that miRNAs like miR-22-3p target specific proteins such as sirtuin 1 (SIRT1), cyclin-dependent kinase inhibitor 1 (p21), methyl-CpG-binding protein 2 (MeCP2), cyclin-dependent kinase inhibitor (CDK1), and phosphatase and tensin homolog (PTEN) (Praticò, 2020; Lauretti et al., 2021). PTEN inhibits the AKT (protein kinase B) signaling pathway, which plays a crucial role in various cellular processes such as cell survival, growth, and metabolism. This suppression leads to increased tau phosphorylation and aggregation. Another example is miR-132-3p, which specifically targets the 3’UTR of the tau protein and controls its phosphorylation and acetylation by targeting enzymes such as EP300, highlighting their role in modulating tau pathology in AD (El Fatimy et al., 2018). MiR-132-3p has been found to target the polypyrimidine tract binding protein 2 (PTBP2), which is involved in tau splicing. Dysregulation of miR-132-3p can thus affect tau splicing and isoform expression. Further, MeCP2, SIRT1, PTEN, and brain-derived neurotrophic factor (BDNF) enhance tau phosphorylation, acetylation, and splicing (Lauretti et al., 2021; Park and Moon, 2022).

Through the interaction with tau protein’s mRNA, miRNAs can induce degradation or inhibit translation, thereby modulating tau protein expression (Silvestro et al., 2019). MiR-132, for example, is a direct regulator of the tau protein. Its deletion may lead to aberrant tau metabolism, increased hyperphosphorylation, and aggregation (Salta et al., 2016). Additionally, miRNAs can also control tau protein expression by targeting other proteins involved in PTM or degradation pathways (Praticò, 2020). MiRNA-125b, for example, has been related to tau hyperphosphorylation and cognitive impairments in AD, presumably via the regulation of other target proteins (Banzhaf-Strathmann et al., 2014). Therefore, miRNAs are crucial for controlling tau expression and its pathological consequences.

The complex relationship between miRNAs and PTMs highlights their importance in AD pathogenesis, providing potential approaches for therapeutic intervention and biomarker discovery to combat this devastating neurodegenerative disorder.

### 5.2 Parkinson disease

Parkinson’s Disease is characterized by protein degradation, protein aggregation, and mitochondrial dysfunction caused by miRNA dysregulation (Lu et al., 2017). In PD, dysregulation of specific miRNAs has been implicated in the modulation of various protein PTMs, including phosphorylation, ubiquitination, acetylation, and sumoylation.

Phosphorylation of proteins such as α-synuclein, tau, and parkin is a hallmark of PD. Various miRNAs control the phosphorylation status of these proteins such as miR-153, miR-7, and miR-124 (Li S. et al., 2016). For instance, miR-153 targets α-synuclein mRNA, reducing its phosphorylation and aggregation, while miR-7 modulates tau protein phosphorylation (Nies et al., 2021). Moreover, miR-124 regulates ubiquitin E3 ligase parkin phosphorylation in PD-associated protein degradation pathways (Kang et al., 2017; Goh et al., 2019; Li et al., 2022).

In Parkinson’s disease, disruptions in ubiquitin-proteasome system (UPS) function have been strongly implicated in the development and progression of the condition. Research indicates that abnormalities in the ubiquitination process can lead to the accumulation of toxic protein aggregates, such as α-synuclein (Walden and Muqit, 2017; Behl et al., 2022; Buneeva and Medvedev, 2022). These protein aggregates can interfere with normal cellular processes, disrupt neuronal function, and ultimately contribute to the degeneration of dopaminergic neurons (Zheng et al., 2016). Evidence of UPS dysfunction in PD includes the presence of ubiquitin-immunopositive Lewy bodies in PD patients’ brains, as well as impaired proteasomal function observed in the substantia nigra of PD brains (Zheng et al., 2016). Multiple miRNAs, such as miR-34b/c, miR-7, and miR-205, target components of the UPS pathway, impacting protein ubiquitination and degradation. For instance, miR-34b/c targets parkin, resulting in its reduced expression and compromised ubiquitin ligase activity (Kabaria et al., 2015; Shlevkov et al., 2016; Jarome and Devulapalli, 2018; Nemeth et al., 2024).

Approximately 25% of miRNA-124 targets showed changed expression patterns in PD. This finding suggests that miRNAs may have multiple targets owing to their poor base pairing with mRNA 3’ UTR (Follert et al., 2014; Lu et al., 2017). In PD brains, miR-9 and miR-124 showed increased expression, and they are thought to regulate genes involved in apoptosis, oxidative stress, and inflammation (Follert et al., 2014; Lu et al., 2017). These results indicate that miRNAs have a significant role in PD pathophysiology. In Table 8, several brain-enriched miRNA families are summarized along with their potential roles in PD pathogenesis.

**Table 8:** Role of microRNA in the pathogenesis of Parkinson’s disease.

miRNA	Role in regulating multiple PD-relevant pathways	References
miR-124	It protects against PD by inhibiting neuroinflammation and apoptosis, affects autophagy, and targets genes in the autophagy-lysosomal pathway.	Jegga et al. (2011), Zhao et al. (2021), and Kanagaraj et al. (2014)
miR-7	It plays a protective role by downregulating α-synuclein by targeting α-synuclein 3′-UTR, modulating mTOR signaling, inhibiting neuroinflammation, promoting glycolysis, and protecting against oxidative stress.	Gao et al. (2002), Doxakis (2010), and Fragkouli and Doxakis (2014)
miR-153	Synergistically represses α-synuclein expression by targeting α-synuclein 3′-UTR and mTOR signaling with miR-7. Sometimes, it impairs the NF-E2-related factor 2 (Nrf2) pathway.	Gao et al. (2002), Doxakis (2010), and Fragkouli and Doxakis (2014)
miR-34	Downregulated in PD, related to mitochondrial function and oxidative stress, upregulates parkin and DJ-1 and might modulate the Nrf2 pathway.	Miñones-Moyano et al. (2011) and Kabaria et al. (2015)
miR-132	The role in PD is not well-established; it may regulate dopamine (DA) neuron differentiation by targeting nuclear receptor-related 1 protein (Nurr1) and play a role in modulating inflammatory responses.	Yang et al. (2012)
miR-133b	Controls the process of dopamine neuron development and the functioning of dopamine neurons, which may impact the expression of α-synuclein.	Hébert and De Strooper (2007) and Kim et al. (2007)
miR-29	Regulates apoptosis, neuronal survival, cellular senescence, motor functions, immune regulation, and epigenetic modulation in PD development.	Smith et al. (2012), Morita et al. (2013), Cao et al. (2018), Chandiran et al. (2018), and Lyu et al. (2018)
miR-485	Potential involvement in various pathways, including apoptosis, immune modulation, synaptic plasticity, and iron homeostasis.	Cohen J.E. et al. (2011), Langenfurth et al. (2014), Wang J. et al. (2018), and Goh et al. (2019)
miR-26	Modulates processes such as apoptosis, DNA repair, autophagy, Long-term potentiation (LTP) maintenance, and immune regulation.	Absalon et al. (2013), Zhao et al. (2014), Gu et al. (2015), Chen C.-Y.A. et al. (2016), Ogino et al. (2016), Jin et al. (2017), Chu et al. (2018), and He et al. (2019)

Unraveling the complex relationship between miRNAs and protein PTMs in PD pathogenesis offers therapeutic avenues. By targeting misregulated miRNAs or their downstream proteins involved in PTMs, there is a potential to restore protein homeostasis and mitigate neurodegeneration in PD patients. Therefore, strategies, including miRNA mimics, antagomirs, and small molecule inhibitors, are being explored to alter miRNA expression or activity.

### 5.3 Amyotrophic lateral sclerosis

Several neurodegenerative disorders, including ALS, are linked to RBP and PTMs. Researchers have identified PTMs, such as phosphorylation, acetylation, ubiquitination, and sumoylation, alter the function, localization, and stability of RBPs. It is suggested that PTMs play an essential role in RBP activities, stress granule dynamics, and the potential for targeting PTMs as a therapeutic intervention in ALS and other neurodegenerative conditions (Lee and Kemper, 2010; Choi and Kemper, 2013). The role of miRNAs in aging and age-related PTM changes has been described in a study examining the biological role and probable significance of m6A RNA methylation in aging and age-related disorders (Sun et al., 2022; Wu et al., 2022).

In ALS, miRNAs play a crucial role in phase transition by promoting or inhibiting the formation of multivalent contacts between phase-separating macromolecules and associating with or excluding other proteins and nucleic acids. These processes can promote or mitigate the proteinopathies that underlie neurodegeneration in ALS (Salem et al., 2022). For example, the PTM of the SOD1 protein, involved in familial cases of ALS, can contribute to disease progression. Changes in the deposition, location, maturation, and modification after translation of the SOD1 protein have been detected in post-mortem spinal cord samples from individuals with ALS (Peggion et al., 2022; Trist et al., 2022). These modifications were linked to instability and incorrect binding of enzymatically active SOD1 dimers, as well as changes to SOD1 modifications after protein synthesis and molecular chaperones that control SOD1 development (Peggion et al., 2022; Trist et al., 2022).

MicroRNAs have been identified as significant contributors to ALS pathogenesis through post-transcriptional gene regulation and interaction with ALS-related proteins. Several miRNAs have been proposed as potential biomarkers for ALS, and their dysregulation has been reported in ALS pathogenesis. Understanding these pathways can lead to novel approaches to ALS treatment and the development of practical diagnostic tools (Salem et al., 2022). Table 9 lists miRNAs involved in ALS pathogenesis. For example, miR-206 regulates myoblast development and maintains neuromuscular connections and synapses (Toivonen et al., 2014). Additionally, it inhibits muscle histone deacetylase 4 (HDAC4), influencing neuromuscular junction re-innervation (Williams et al., 2009). ALS patients exhibit a significant increase in miR-23 levels. It inhibits the translation of proliferator-activated receptor γ coactivator 1 α (PGC-1α) (a receptor activated by peroxisome proliferators and involved in mitochondria biosynthesis and functioning) (Russell et al., 2013). A miR-193b-3p controls the mechanistic target of rapamycin complex 1 (mTORC1) activity by targeting tuberous sclerosis 1 (TSC1), influencing cell survival and autophagy mechanisms (Li C. et al., 2017). The study found that miR-183b-5p targets RIP kinase 1 (RIPK) and programmed cell death 4 (PDCD4), indicating its role in programmed neuronal cell death (Li C. et al., 2017). A miR-155 is implicated in ALS neuroinflammation, and binding to SOCS1 mRNAs is linked to a rise in pro-inflammatory cytokines (Ceppi et al., 2009; O’Connell et al., 2010). These studies indicate that miRNAs play an essential role in ALS pathogenesis.

**Table 9:** Role of miRNA in the pathogenesis of amyotrophic lateral sclerosis.

miRNA	Role in ALS pathologies	References
miR-125b	The A20 protein is a protective mechanism against the death of MNs (motor neurons) caused by activated G93A microglia by suppressing miR-125b.	Parisi et al. (2013, 2016)
miR-375-3p	Downregulating the expression of miR-375-3p leads to ineffective regulation of p53, causing an increase in the production of NDRG2. This, in turn, produces reactive oxygen species, initiating a damaging loop.	Rohm et al. (2019)
miR-18b-5p	The presence of miR-18b-5p led to the activation of HIF1α, resulting in an upregulation of Mef2c expression. Mef2c upregulated the transcription factor miR-206 expression. The suppression of mctp1 and RARB, targeted by miR-206, resulted in increased levels of intracellular Ca2+ and decreased cell differentiation, respectively.	Kim et al. (2020)
miR-124	The increase in miR-124 expression is associated with the deterioration of mSOD1 motor neurons, the disruption of communication between the nervous and immunological systems, and the disturbance of internal stability.	Vaz et al. (2021)
miR-9 and miR-105	Downregulation of miR-9 and miR-105 in ALS could disrupt the balance of intermediate filaments, ultimately leading to the clumping of these filaments and, ultimately, the death of neurons.	Hawley et al. (2019)
miR-126-5p	Downregulation promoted axon degeneration and the neuromuscular junction (NMJ) breakdown.	Maimon et al. (2018)
miR-1825	Downregulation increases the production of TBCB (tubulin-folding cofactor b), which can potentially trigger the breakdown and degradation of TUBA4A (tubulin α-4A chain)	Helferich et al. (2018)

The research findings in the search support the significant impact of PTMs and miRNAs on ALS pathogenesis. Both PTMs and miRNAs are implicated in the dysregulation of proteins and gene expression associated with ALS, highlighting their potential as critical regulatory mechanisms and offering potential avenues for therapeutic intervention and diagnostic development. As a result, further research is needed into the role of PTMs and miRNAs in ALS.

### 5.4 Huntington diseases

Huntington’s Disease pathogenesis has been linked to protein PTMs, such as those of mHTT, a neuronal signaling protein, and other proteins involved in neuronal signaling (Lontay et al., 2020). As post-transcriptional regulators, miRNAs can indirectly influence these PTMs by regulating the expression of enzymes that carry out the modifications or proteins that target those modifications. In huntingtin protein, specific miRNAs can bind to the mRNA and inhibit it from being translated, thereby affecting levels of mHTT and PTMs that follow (Martinez and Peplow, 2021). Although it is not entirely understood what exactly miRNAs influence PTMs in HD, several miRNAs whose expression levels are altered, have been identified in patients with HD, including miR-1247-5p, miR-10b-5p, miR-615-3p, miR-196b-5p, and miR-196a-5p (Hoss et al., 2014; Guo et al., 2022). By overexpressing miR-196a and miR-155 in animal models of HD, mHTT mRNA, and protein levels decreased, indicating that these miRNAs could influence mHTT PTMs by regulating their expression (Martinez and Peplow, 2021). Table 10 provides a list of miRNAs associated with HD pathogenesis.

**Table 10:** Role of miRNA in the pathogenesis of Huntington’s disease.

miRNA	Role in HD	References
miR-9	It is downregulated in HD and is associated with transcriptional dysregulation. It targets genes that play essential roles in HD pathophysiology.	Packer et al. (2008)
miR-10b-5p	Upregulated in HD, potentially promoting striatal involvement in the disease. In normal circumstances, it controls the level of BDNF.	Hoss et al. (2015a)
miR-146a	Exerts a negative regulatory effect on the NF-κB pathway, and its expression is diminished in HD. It specifically targets genes that regulate the cell cycle and apoptosis, perhaps aiding in correcting anomalies in these processes.	Sinha et al. (2010)
miR-196a	It is upregulated in HD and is associated with reduced cytotoxicity and apoptosis. It improves mitochondrial function and morphology by upregulating essential genes like CBP and PGC-1α.	Cheng et al. (2013) and Fu et al. (2015)
miR-214	Upregulated and targets the HTT gene. It may influence the aggregation of mHTT and impact mitochondrial morphology and cell cycle regulation.	Bucha et al. (2015) and Dong and Cong (2021)

There is evidence that miRNAs such as miR-128 and miR-155 play a role in regulating phosphorylation events related to HD pathology. It is reported that miR-128 targets the mRNA of p35, a regulatory subunit of CDK5 (Guzman et al., 2018; Lian et al., 2018; Budi et al., 2023) Similarly, miR-155 targets the mRNA of glycogen synthase kinase 3 beta (GSK3β), a key kinase involved in mHTT phosphorylation and toxicity (Haramati et al., 2010; Shi et al., 2012; Varendi et al., 2014; Budi et al., 2023).

Impaired UPS function contributes to misfolded protein accumulation in HD. MicroRNAs such as miR-9 and miR-27a have been implicated in regulating UPS components, influencing protein ubiquitination and degradation. The miR-9 targets the mRNA of ubiquitin-conjugating enzyme E2I (UBE2I), while miR-27a targets the mRNA of ubiquitin-specific protease 25 (USP25), both involved in UPS-mediated protein degradation (Ma et al., 2010; Zhang et al., 2019; Dong and Cong, 2021; Zhu et al., 2021).

Acetylation of proteins by histone acetyltransferases (HATs) and HDACs plays a role in HD development. It has been associated with miRNAs that modulate acetylation processes, such as miR-22 and miR-124. The miR-22 targets the mRNA of HDAC4, a class II HDAC involved in mHTT-induced neurotoxicity, while miR-124 targets the mRNA of HAT1, a histone acetyltransferase implicated in transcriptional dysregulation in HD (Lee et al., 2011; Liu et al., 2020; Dong and Cong, 2021; Zhang et al., 2023).

MicroRNAs such as miR-132 and miR-146a have been implicated in modulating SUMOylation processes in HD. It is reported that miR-132 targets the mRNA of SUMO1, while miR-146a targets the mRNA of SUMOylation enzyme protein inhibitor of activated STAT1 (PIAS1), both of which regulate SUMOylation-mediated protein interactions and cellular homeostasis (Qian et al., 2017; Fan et al., 2020; Acuña et al., 2023).

In summary, studying miRNAs and abnormal proteins in HD is an active research area. This may provide a better understanding of disease mechanisms and therapeutic targets. It is critical to conduct further research on miRNAs and abnormal proteins to understand better their role and potential implications in Huntington’s disease. In addition, it is critical to develop effective biomarkers to monitor HD progression and treatment response.

### 5.5 Multiple sclerosis

Multiple Sclerosis is characterized by a significant involvement of miRNAs in pathophysiology, primarily affecting glial and peripheral immune cells (Gao et al., 2021). MicroRNAs mediate several cellular functions and developmental pathways, exhibiting various expression patterns. In addition to regulating T lymphocytes, they also play a role in MS development and progression (O’Brien et al., 2018; Michlewski and Cáceres, 2019; Soni and Biswas, 2021; Velázquez-Cruz et al., 2021). The dysregulation of miRNA can result in abnormal T lymphocyte function, potentially contributing to the autoimmune nature of MS. According to studies, miRNAs regulate T-cell-mediated immunity, an important aspect of MS pathophysiology (Wang and Liang, 2022). To develop new therapeutic strategies for MS, it is necessary to understand the role of miRNAs on T lymphocyte function.

Their effects on gene expression can explain the role of miRNAs in PTM. A critical role for miRNAs in MS is to modulate glial cells, another important player. We can gain a better understanding of immune-related disorders when we understand how miRNAs function in these processes. A majority of lesions (active and inactive), as well as normal-appearing white matter (NAWM), upregulate miR-155 (O’Brien et al., 2018; Michlewski and Cáceres, 2019; Soni and Biswas, 2021; Velázquez-Cruz et al., 2021). It regulates macrophage responses to inflammatory stimuli and impacts macrophage activity, which can indirectly influence PTM by modulating macrophage interactions with other proteins (Maciak et al., 2021; Zingale et al., 2022). MS patients have lower miR-320a expression and higher matrix metallopeptidase-9 (MMP-9) expression. MMP-9 disrupts the blood–brain barrier and degrades the essential myelin protein, contributing to the pathogenesis of MS. While MMP-9 is involved in protein degradation (a PTM-related process), miR-320a’s primary role is regulating gene expression (Aung et al., 2015). The miR-124 influences the activity of macrophages and microglial cells by targeting transcription factors (Ponomarev et al., 2011). MiR-338 is upregulated in NAWM and targets neurosteroid synthesis enzymes, potentially leading to PTM by altering the levels of specific molecules (Noorbakhsh et al., 2011; de Faria et al., 2013). MiR-23a plays a part in the differentiation of oligodendroglia and the myelination process, suggesting it plays a role in PTM by influencing processes related to myelination (Lin et al., 2013). The transformation of neural progenitor cells into oligodendrocytes promotes new neuron development. It reduces the formation of oligodendrocyte precursor cells, and peripheral myelin protein synthesis is suppressed by miR-9 regulation (Zhao et al., 2009; Coolen et al., 2013; Suzuki et al., 2020). MiR-27a has a crucial role in generating mature oligodendrocytes and influencing myelination. Its role in myelination suggests a potential role in PTM related to myelin proteins (Tripathi et al., 2019).

Although some of these miRNAs are not directly implicated in PTM, their regulatory effects on various cellular processes can indirectly affect PTM (Table 11). According to a study, search results provide more insight into the relationship between miRNAs and PTMs (Visser and Thomas, 2021). Micro RNAs may be candidates to control DNA damage response (DDR) enzyme levels and PTM. Histone PTM can also enhance or repress miRNA expression. The interplay between transcription factors and miRNAs can also profoundly impact gene expression, influencing PTM (Martinez and Walhout, 2009; Morales et al., 2017). As a result of these studies, cellular processes, including PTM, could be influenced by miRNAs via various regulatory mechanisms. This could provide insights into the intricate interactions between miRNAs and cellular processes.

**Table 11:** Role of miRNA in the pathogenesis of multiple sclerosis.

miRNA	Role in MS pathogenesis	References
miR-124	It is downregulated and functions in cell differentiation, particularly in the control of CCAAT/enhancer-binding protein (CEBP), which is associated with the formation of myeloid cells.	Veremeyko et al. (2019) and Zhang et al. (2023)
miR-155	It contributes to releasing inflammatory factors by activating microglia and causing inflammation.	Moore et al. (2013), Alivernini et al. (2018), and Zingale et al. (2022)
miR-219	Exosomes obtained from young and environmentally enriched (EE; volitionally increased intellectual, social, and physical activity) animals have a high concentration of miR-219, which is essential and adequate for generating myelinating oligodendrocytes. It is achieved by lowering the expression of differentiation inhibitory regulators.	Pusic and Kraig (2014)
miR-17-5p	It increases expression and affects the regulatory subunit 1 of phosphatidylinositol 3-kinase (PI3K) and PTEN, which are involved in post-translational modification activities.	Lindberg et al. (2010)
miR-125a-3p	Upregulation inhibits remyelination, indicating involvement in PTM processes related to myelin maintenance.	Lecca et al. (2016) and Marangon et al. (2020)
miR-873	Upregulation activates the NF-κB and secretes the inflammatory molecules in astrocytes, contributing to neuroinflammation in MS.	Liu et al. (2014) and Long et al. (2020)
miR-223	MiR-223 has a mixed regulatory pattern and may also influence PTM processes in MS pathology.	Li et al. (2018), Morquette et al. (2019), and Jiao et al. (2021)

## 6 MicroRNAs in aging

MicroRNAs regulate many genetic processes and biological pathways during aging. Their fluctuations in body fluids are associated with aging and age-related diseases such as NDs. In mammalian tissues, including the heart, brain, muscles, and bones, they have been demonstrated to regulate age-related processes and pathologies. Additionally, miRNAs regulate longevity in invertebrates through canonical aging pathways in invertebrates such as *Drosophila* and *Caenorhabditis elegans* (Santos and Lindner, 2017; Kinser and Pincus, 2020). Researchers demonstrated that mammalian brain miRNA composition changes with normal aging (Li N. et al., 2011; Inukai et al., 2012; Yin et al., 2015; Chen et al., 2019).

Further, miRNA expression changes are specific to certain brain regions (Persengiev et al., 2012). It is worth noting that the intricacies of normal brain aging remain complex and not yet fully understood. Consequently, the primary focus of miRNA research has been its role in age-related neuropathologies. Next, we highlight some evidence regarding the involvement of miRNAs in the brain’s aging.

- MiR-34 in Drosophila: It is upregulated induced by aging and is essential for brain health. Mutants of this miRNA show accelerated brain aging, while its upregulation extends lifespan and improves neurodegeneration (Liu et al., 2012).
- MiR-1000 in Drosophila: MiR-1000 plays a vital role in neuroprotection in aging by regulating glutamate transporters—mutants of miR-1000 experience shorter lifespans and neurodegeneration due to disrupted glutamate homeostasis (Verma et al., 2015).
- MiR-34 in Mammals: This miRNA is upregulated in the aging of AD patients. Its overexpression in the mouse hippocampus leads to memory impairment and decreased SIRT1 expression, a lifespan regulator. In mouse brain and blood, miR-34a levels increase with age and are inversely related to SIRT1 expression (Li et al., 2011b; Zovoilis et al., 2011).
- MicroRNAs and calorie restriction: Age-dependent miR-34a upregulation does not occur in calorically rested rodent brains. In calorie-restricted rodents, age increases the expression of the anti-apoptotic gene B-cell lymphoma 2 (BCL-2), targeted by miR-34a (Khanna et al., 2011).
- MiR-29 family: MiR-29a/b are upregulated with age in the mouse brain, contributing to microglia dysregulation and neuroinflammation, standard features of brain aging. These miRNAs directly suppress IGF-1 and CX3CL1 inhibitors of microglia activity (Fenn et al., 2013; Takeda and Tanabe, 2016; Ripa et al., 2017).

Studies have shown variations in miRNA expression patterns linked to NDs and prion-induced neurological conditions (Junn and Mouradian, 2012). The miRNA let-7, which targets the signaling of the nuclear receptor DAF-12 (abnormal dauer formation protein 12) and influences lifespan in worms (Junn and Mouradian, 2012), has been associated with AD. This relationship is made clear through genetic interactions observed in worms between let-7 and the homolog of APP known as APP-like-1 (apl-1). According to these findings, miRNA regulation may be involved in controlling Aβ peptide formation, even in non-mammalian organisms (Wang et al., 2008; Provost, 2010). In addition, miR-320 targets IGF-1 and IGF-1R in rats (Baek et al., 2008) and is upregulated in prion-induced neurological disorders (Saba et al., 2008). Table 12 lists dysregulated miRNAs for aging in human body fluids.

**Table 12:** List of dysregulated miRNA to aging in human body fluids.

Sample	miRNA	Expression	Method	References
Serum	miR-222, miR-92a, miR-375	Upregulated	Solexa sequencing, RT-qPCR	Zhang et al. (2015)	miR-142-5p, miR-130b, miR-29b, miR-340	Downregulated	Solexa sequencing, RT-qPCR	Eschenhagen and Zimmermann (2005) and Catanesi et al. (2020)	miR-181a-5p, miR-151a-3p, miR-1248.	Downregulated	Illumina sequencing, RT-qPCR	Hooten et al. (2013)	miR-20a	Downregulated	RT-qPCR	Sawada et al. (2014)	miR-1225-3p, miR-211-5p, miR-5095	Upregulated	RT-qPCR	Smith-Vikos and Slack (2012)	miR-340, miR-374a-5p, miR-376c-3p	Downregulated	RT-qPCR	Gerasymchuk et al. (2020) and Eshkoor et al. (2022)
Plasma	miR-142-3p, miR-126-3p, let-7a-5p, miR-30b-5p, miR-30c-5p, miR-210	Upregulated	RT-qPCR	Ameling et al. (2015)	miR-93-5p	Downregulated	RT-qPCR		miR-126-3p	Upregulated	RT-qPCR	Olivieri et al. (2014)	miR-619, miR-3615	Downregulated	Illumina sequencing	Wu S. et al. (2016)
Saliva	miR-24-3p	Upregulated	Microarray, RT-qPCR	Machida et al. (2015)
Whole blood	miR-1284, miR-1262, miR-145-5p, miR-93-3p, miR-34a-5p	Upregulated	Microarray and Illumina sequencing	Meder et al. (2014)

In conclusion, miRNA dysregulations in various aging-related disorders support the idea that aging is a fundamental factor in developing age-related diseases. MicroRNAs in body fluids fluctuate with age and age-related diseases. However, they could be used as potential biomarkers for aging, longevity, and monitoring the effectiveness of interventions to reduce the rate of aging. Investigations have examined changes in miRNA expression in various body fluids concerning age, including serum, plasma, and saliva (Smith-Vikos et al., 2016; Kinser and Pincus, 2020). A miRNA’s expression is regulated up or down with age, with some miRNAs playing a role in inflammation, cancer, cell cycle regulation, and DNA repair. A few studies have attempted to link circulatory miRNA profiles directly to human lifespan, showing associations between specific miRNAs and longevity (Caravia and López-Otín, 2015; Smith-Vikos et al., 2016; Kinser and Pincus, 2020; Zia et al., 2022). These studies have low overlap, and the hunt for a clear miRNA biomarker of aging remains due to research design, profiling methodologies, and individual backgrounds. Although miRNA expression patterns are inconsistent, they offer valuable insight into aging and its progression (Hooten et al., 2013; Olivieri et al., 2014; Sawada et al., 2014; Ameling et al., 2015; Machida et al., 2015; Zhang et al., 2015).

### 6.1 Summary and future perspectives

MicroRNA dysregulation has been extensively studied in NDs. Several studies have investigated miRNA dysregulation within specific NDs (Brites, 2020; Proshkina et al., 2020), while others examined miRNAs across multiple NDs (Brites, 2020; Proshkina et al., 2020). A study found that miR-155-5p, miR-146a-5p, and miR-223-3p were upregulated in patient specimens and animal models of 12 neurodegenerative diseases, such as AD, PD, HD, ALS, and MS, using Reporting Items for Systematic Reviews and Meta-Analyses (PRISMA). However, miR-132-3p, miR-21-5p, miR-29, miR-9-5p, and miR-124-3p exhibit mixed regulation (Juźwik et al., 2019). MiRNA dysregulation has been demonstrated in NDs, but questions remain regarding their suitability as diagnostic markers. MiRNAs can pose challenges when used as specific diagnostic markers due to their complex pathological characteristics and overlapping expression patterns between different NDs.

Our review delved into the landscape of miRNA biomarkers across various NDs, encompassing AD, PD, ALS, HD, and MS. Through meticulous examination of miRNA profiles across diverse sample sources, including serum, plasma, CSF, blood, and brain tissue, we aimed to elucidate unique miRNA signatures indicative of each disease’s pathogenesis. Our analysis unveiled a diverse array of dysregulated miRNAs across different sample types. For instance, in AD, miR-135a and miR-384 consistently exhibited upregulation (Table 1), whereas PD showcased downregulation of miR-146a-5p and miR-132-3p (Table 2). Additionally, ALS demonstrated consistent upregulation of miR-338-3p (Table 3), while HD exhibited upregulation of miR-34b (Table 4). Many miRNAs were upregulated in MS, including miR-223, miR-148b, miR-24, miR-34b, and miR-324-3p (Table 5). Moreover, several miRNAs displayed disease-specific dysregulation, potentially serving as diagnostic biomarkers or therapeutic targets.

Interestingly, miR-124 exhibited consistent downregulation across all diseases, while miR-146a and miR-223 had mixed regulation, suggesting that these genes play a crucial role in pathogenesis. An important point to emphasize is that a study by Mancuso et al. (2019) demonstrated that miRNAs can distinguish between several NDs, including AD and PD. According to them, miR-223 expression levels can be a discriminatory factor between these groups. This study has shown that miRNA dysregulation is associated with various NDs. For effective treatment and management strategies, it is crucial to identify disease-specific and shared changes in miRNA expression (Mancuso et al., 2019). However, despite promising results, it is necessary to conduct additional research to determine the accuracy and reliability of miRNAs for identifying various neurodegenerative diseases (Viswambharan et al., 2017; Wang and Zhang, 2020; Gentile et al., 2022; Nguyen et al., 2022; Li et al., 2023).

Overall, miRNA expression in NDs is influenced by many factors, including disease type and sample origin, producing a complex picture. The detailed analysis of our data reveals a diverse landscape of dysregulated miRNAs across different NDs. In our study, miRNA-9, miR-146a, and miR-181c emerged as prominent candidates with recurrent expression level changes across multiple diseases. In addition, miR-29a, miR-132, and miR-133b are consistently dysregulated, indicating ND pathogenesis. Our findings provide valuable insights into the complex mechanisms underlying NDs, despite the need for further investigation to clarify their precise roles in disease pathogenesis. The findings pave the way for future research projects examining miRNA-mediated molecular pathways in neurodegeneration.

It is challenging to accurately evaluate miRNA data due to the technical limitations of methods such as RT-qPCR and NGS (Huggett et al., 2013). These challenges can lead to inconsistent results, hindering miRNA expression assessment in NDs. Further complicating miRNA data interpretation in NDs is the lack of reliability and consistency in bioinformatics and biostatistical methods used to analyze miRNA expression data (Tellinghuisen and Spiess, 2019). Therefore, miRNA expression in NDs may not be consistent across studies.

MicroRNAs and PTMs in NDs are an emerging area of research. MicroRNAs influence PTMs indirectly by regulating the expression of enzymes that perform these modifications or proteins that target these modifications. MicroRNAs and PTMs enhance the regulatory mechanism for NDs. However, the specific impact of miRNAs on PTMs remains to be determined, and further research is needed. The technical challenge of measuring miRNA expression levels accurately in NDs is the need for specific and susceptible technologies, such as RT-qPCR and NGS. A further challenge in miRNA expression bioinformatics is identifying relevant miRNA targets, processing data, and normalizing it. Measurement and interpretation of miRNA expression data in the context of NDs require attention to these technical challenges.

In summary, miRNAs have shown great promise as diagnostic biomarkers for NDs. To advance this emerging field of research, it is critical to identify and characterize new miRNAs implicated in NDs. A critical need still exists for further research into how miRNAs influence various NDs, even though advancements in this field have been significant. However, further research is necessary to comprehend the complex regulatory roles of miRNAs fully. This is to overcome technical difficulties in measuring and analyzing them accurately. It is also to optimize their utility as diagnostic tools and treatment options within clinical contexts.

## Author contributions

HA: Writing – original draft, Writing – review & editing, Conceptualization, Data curation, Formal analysis, Investigation, Methodology, Visualization. RR: Writing – review & editing. CG: Writing – review & editing. MK: Writing – review & editing. FD: Writing – review & editing. KH: Writing – review & editing. HP: Writing – review & editing. BH: Writing – review & editing. DR: Writing – review & editing. PS: Writing – review & editing, Conceptualization, Data curation, Formal analysis, Funding acquisition, Investigation, Methodology, Project administration, Resources, Software, Supervision, Validation, Visualization. SR: Conceptualization, Data curation, Formal analysis, Funding acquisition, Investigation, Methodology, Project administration, Resources, Software, Supervision, Validation, Visualization, Writing – review & editing.

## Figures and Tables

**Table:** 

Aβ	Amyloid beta
Ac-Tau	Acetylation tau
AD	Alzheimer’s disease
ADAM	A disintegrin and metalloproteinase
AGO2	Argonaute RISC Catalytic Component 2
AKT	Protein kinase B (“AK” AKR mouse strains; T thymoma)
ALS	Amyotrophic lateral sclerosis
APP	Amyloid beta precursor protein
BACE1	β-site amyloid precursor protein-cleaving enzyme 1
BDNF	Brain-derived neurotrophic factor
CNS	Central nervous system
CSF	Cerebrospinal fluid
CX3CL1	C-X3-C motif chemokine ligand 1
DGCR8	DiGeorge critical region 8
FTD	Frontotemporal dementia
GABA	Gemma (γ)-aminobutyric acid
GSK3	Glycogen synthase kinase 3β
HD	Huntington’s disease
Htt	Huntingtin
IGF-1	Insulin-like growth factor
MeCP2	Methyl-CpG binding protein 2
mHTT	Mutant huntingtin protein
mRNA	Messenger RNA
miRNA	Micro RNA
miRNPs	Micro-ribonucleoprotein
miRNPs	Micro-ribonucleoprotein
MMP-9	Matrix metallopeptidase-9
MRI	Magnetic resonance imaging
MS	Multiple sclerosis
NAWM	Normal-appearing white matter
ND	Neurodegenerative disease
NfL	Neurofilament light chain
NFT	Neurofibrillary tangles
NF-κB	Nuclear factor kappa-light-chain-enhancer’ of activated B-cells
NGS	Next generation sequencing
NMRS	Nuclear magnetic resonance spectroscopy
Nrf2	Nuclear factor erythroid 2-related factor 2
PD	Parkinson’s disease
PET	Positron emission tomography scan
PGC-1α	Proliferator-activated receptor γ coactivator 1 α
PI3K	Phosphoinositide 3-kinases
pre-miRNA	Precursor microRNA
PTEN	Phosphatase and tensin homolog
PTM	Post-translational modification
RT-qPCR	Quantitative reverse transcription polymerase chain reaction
RBP	RNA binding proteins
RISC	RNA-induced silencing complex
siRNAs	Small interfering RNAs
SIRT1	Sirtuin-1
SOD1	Superoxide dismutase
UMN	Upper motor neuron
UVR	Ultraviolet radiation
